# The 1995 North American Interagency Intercomparison of Ultraviolet Monitoring Spectroradiometers

**DOI:** 10.6028/jres.103.002

**Published:** 1998-02-01

**Authors:** Edward Early, Ambler Thompson, Carol Johnson, John DeLuisi, Patrick Disterhoft, David Wardle, Edmund Wu, Wanfeng Mou, Yongchen Sun, Timothy Lucas, Tanya Mestechkina, Lee Harrison, Jerry Berndt, Douglas S. Hayes

**Affiliations:** National Institute of Standards and Technology, Gaithersburg, MD 20899-0001 USA; National Oceanic and Atmospheric Administration, R/E/ARx1, 325 Broadway, Boulder, CO 80303 USA; Atmospheric Environment Service, Environment Canada, 4905 Dufferin Street, Toronto, ON M3H 5T4, Canada; Dept. of Physics and Astronomy, University of Georgia, Athens, GA 30602 USA; Biospherical Instruments Inc., 5340 Riley Street, San Diego, CA 92110-2621 USA; Atmospheric Sciences Research Center, State University of New York Albany, 100 Fuller RD, Albany, NY 12205 USA; Smithsonian Environmental Research Center of the Smithsonian Institution, P.O. Box 28, Edgewater, MD 21037 USA

**Keywords:** environmental monitoring, intercomparison, solar ultraviolet, spectroradiometers

## Abstract

Concern over stratospheric ozone depletion has prompted several government agencies in North America to establish networks of spectroradiometers for monitoring solar ultraviolet irradiance at the surface of the Earth. To assess the ability of spectroradiometers to accurately measure solar ultraviolet irradiance, and to compare the results between instruments of different monitoring networks, the second North American Intercomparison of Ultraviolet Monitoring Spectroradiometers was held June 12 to 23, 1995 at Table Mountain outside Boulder, Colorado, USA. This Intercomparison was coordinated by the National Institute of Standards and Technology (NIST) and the National Oceanic and Atmospheric Administration (NOAA). Participating agencies were the Environmental Protection Agency; the National Science Foundation; the Smithsonian Environmental Research Center; the Department of Agriculture; and the Atmospheric Environment Service, Canada. Instruments were characterized for wavelength uncertainty, bandwidth, stray-light rejection, and spectral irradiance responsivity, the latter with a NIST standard lamp operating in a specially designed field calibration unit. The spectral irradiance responsivity, determined once indoors and twice outdoors, demonstrated that while the responsivities changed upon moving the instruments, they were relatively stable when the instruments remained outdoors. Synchronized spectral scans of the solar irradiance were performed over several days. Using the spectral irradiance responsivities determined with the NIST standard lamp and three different convolution functions to account for the different bandwidths of the instruments, the measured solar irradiances generally agreed to within 3 %.

## 1. Introduction

Networks of spectroradiometers for monitoring solar ultraviolet irradiance at the surface of the Earth have been established by several government agencies in North America in response to concern over stratospheric ozone depletion. Detecting long-term trends in solar ultraviolet irradiance requires accurate measurements of the absolute irradiance for individual instruments, for the entire network, and between networks [1].

To assess the ability of spectroradiometers to accurately measure solar ultraviolet irradiance, and to compare these results between instruments of different monitoring networks, North American Interagency Intercomparisons of Ultraviolet Monitoring Spectroradiometers have been performed outside Boulder, Colorado. The first such Intercomparison was held September 19 to 29, 1994, and the experimental details and results from this effort are described in Ref. [2]. Results from the second Intercomparison, held June 12 to 23, 1995, are presented here. This Intercomparison was coordinated by the Optical Technology Division of the National Institute of Standards and Technology (NIST) and the Surface Radiation Research Branch (SRRB) of the National Oceanic and Atmospheric Administration (NOAA). Spectroradiometers from monitoring networks administered by the following agencies participated: the Environmental Protection Agency (EPA), the National Science Foundation (NSF), the Department of Agriculture (USDA), the Smithsonian Environmental Research Center of the Smithsonian Institution (SERC), and the Atmospheric Environment Service (AES) of Canada. A list of attendees is given in Appendix A.

While one goal of the second Intercomparison was simply to reproduce the measurements performed at the first, there were several substantial improvements in experimental techniques from the first to the second. The variety of spectroradiometers increased, with one fewer Brewer instrument and the addition of an instrument from the USDA. The second Intercomparison was purposely held during the summer solstice in an attempt to maximize the solar ultraviolet irradiance. The same instrument parameters were characterized at both Intercomparisons, namely wavelength uncertainty, stray-light rejection, the slit-scattering function, and spectral irradiance responsivity, but at the second all characterizations were performed both indoors and outdoors. This was done to assess the validity of the experimental techniques used outdoors. In particular, a new field calibration unit was used for the spectral irradiance responsivity measurements, which dramatically improved the technique for this experiment.

The spectral irradiance responsivity both checked the absolute irradiance scales used by the networks and provided a common scale for the synchronized measurements of solar irradiance. As at the first Intercomparison, these synchronized measurements were the most important aspect of the second Intercomparison because they assess the present limits to which irradiances determined by different instruments can be compared. Other instruments determined the atmospheric conditions during the Intercomparison, which will be useful for correlating these conditions with the measured solar ultraviolet irradiance. A list of all the instruments present at the Intercomparison is given in Table 1.1. Note that all times given in this paper are in Universal Coordinated Time (UTC), which was 6 h ahead of Mountain Daylight Time, the local time.

## 2. Site Description

The site of the Intercomparison was Table Mountain, a plateau owned by the Federal Government approximately 12.9 km north of Boulder, Colorado and 5.6 km east of the front range of the Rocky Mountains. This site was chosen because of its good view to the horizon, the presence of laboratory facilities, and the proximity of facility and staff support at both NIST and NOAA in Boulder.

For the synchronized measurements of solar irradiance, the spectroradiometers were located on individual concrete pads on the south side of the plateau at latitude 40.125° N, longitude 105.237° W, and elevation 1689 m. The pads were arranged in an east-west line and were 2.4 m square with 12.2 m between centers. The highest, and only major, obstruction to the horizon was a peak 5.6 km due west of the pads with a 5.1° angle of inclination. Temporary trailers approximately 30 m south of the pads housed the data acquisition and control computers and equipment for the spectroradiometers. The plateau sloped downward south of the pads, so the tops of the trailers were below the elevation of the pads. A test facility platform approximately 30 m west of the west-most pad is NOAA’s SRRB site. At the Intercomparison site, pyranometers, pyrgeometers, radiometers, and shadowband radiometers were located on the platform. A meteorological tower recording the temperature, relative humidity, atmospheric pressure, and wind speed and direction at the site was located approximately 90 m northwest of the pads. Finally, a concrete building immediately to the southwest of the platform was used for servicing the instruments, holding meetings, and performing indoor characterizations. A dome at the western end of the building was covered with a black cloth to eliminate reflections from it to the instruments.

## 3. Instrument Descriptions

Five instruments participated at the Intercomparison. A Brewer Spectrophotometer^1^, Model MKII, serial number 039, was operated by the participants from AES Canada. The instrument from the EPA network was also a Brewer Spectrophotometer, Model MKIV, serial number 114, and was operated by participants from the University of Georgia, who manage the EPA network. The NSF instrument was a Biospherical Instruments SUV-100 Ultraviolet Spectroradiometer, serial number B-007, operated by participants from that company, who also administer the NSF network. Participants from SERC operated a Smithsonian SR-18 Ultraviolet Scanning Radiometer, serial number UD. The instrument from the USDA network was an Ultraviolet Spectroradiometer developed by the Atmospheric Science Research Center (ASRC) at the State University of New York (SUNY), Albany and Research Support Instruments, Inc., serial number 901, and was operated by participants from ASRC. For the remainder of this paper, these instruments will be designated AES, EPA, NSF, SERC, and USDA, respectively. With the exception of the SERC instrument, the spectroradiometers operate by scanning a specified wavelength range. The signal is measured at discrete wavelengths, from shortest to longest separated by a fixed interval, within a specified range. Table 3.1 lists the characteristics of each instrument, and descriptions are given below.

### 3.1 Brewer Spectrophotometer

The Brewer Spectrophotometer measures total solar ultraviolet irradiance from 290 nm to 325 nm (Model MKII) or from 286.5 nm to 363 nm (Model MKIV) and total column O_3_ and SO_2_ from both direct sun and zenith sky measurements at specific ultraviolet wavelengths. The Model MKIV also determines total column NO_2_. A right-angle prism directs light from one of several sources—either internal calibration lamps, the sky, or a Teflon diffuser—along the optical path. This path contains apertures, filters, and lenses which focus the light onto the entrance slit of a single-grating modified Ebert-type monochromator.

The exit slit focal plane of the monochromator contains six slits, five for selecting the wavelengths for determining the total column O_3_ and SO_2_ and one for wavelength calibration. A slotted cylindrical slitmask in front of the exit slit plane serves as the wavelength selector. The nominal bandwidth, set by the exit slits, is 0.6 nm. For a Model MKII, the diffraction grating operates in third order and the first slit is selected, while the extended wavelength range of the Model MKIV is achieved by changing to second order and a different exit slit at 325 nm.

Light from the exit slit passes through a lens and a filter before focusing onto the cathode of a photomultiplier tube (PMT). The electrical pulses, generated by photons, from the PMT are amplified, discriminated, and divided by four before being transmitted to the counter. In the MKII model, the filter is NiSO_4_ sandwiched between two Schott UG-11 filters. The MKIV model has the same filter for wavelengths shorter than 325 nm, and a single UG-11 filter for longer wavelengths.

The wavelength of the monochromator in terms of micrometer steps was determined at the factory from the wavelengths of Hg emission lines. The wavelength registration of the monochromator is periodically checked and adjusted throughout a day by scanning the micrometer forward and backward about the 302.3 nm line from the internal Hg calibration lamp.

The two networks, AES and EPA, use different procedures for determining the spectral irradiance responsivity of their instrument from their spectral irradiance scale. The AES uses 1000 W DXW-type quartz-halogen lamps operating in the horizontal position 40 cm above the diffuser. The lamp is housed in a custom enclosure with air drawn over the lamp, and baffling limits the light falling on the diffuser to the direct beam from the lamp. The current from a power supply is monitored through a calibrated shunt resistor by a voltmeter so that the operator can manually adjust the current as needed. The EPA uses the set of calibration lamps, housing, and power supply furnished by the manufacturer. These are 50 W quartz-halogen lamps mounted horizontally 5 cm above the diffuser in a housing and operated at a constant 12 V.

### 3.2 Biospherical Ultraviolet Spectroradiometer

The Biospherical SUV-100 B-007 Ultraviolet Spectroradiometer measures total solar irradiance from 280 nm to 620 nm. One beamsplitter directs light from either of two internal calibration sources onto a second beamsplitter. This second beamsplitter directs either this beam or one from a Teflon diffuser onto the entrance slit of a double-grating monochromator. There is a quartz relay lens between the diffuser and the second beamsplitter.

The light from the exit slit of the monochromator is detected by a PMT operating in current mode. The high voltage applied to the PMT is variable and for a specific spectral scan is set to obtain maximum sensitivity. The PMT is mounted in a shielded housing and operated at 20 °C while the temperature of the monochromator is controlled to 32.5 °C. The bandwidth of the instrument is nominally 0.95 nm.

One of the internal calibration sources is a Hg emission lamp. The lines from this lamp are scanned several times each day, and are used to register the monochromator at the 296.7 nm line and determine the wavelength calibration. The other internal calibration source is a 45 W lamp for determining the spectral irradiance responsivity of the instrument. The responsivity is determined in a two-step process since the high voltage of the PMT is variable. The spectral irradiance scale is realized by a 200 W DXW-type quartz-halogen lamp mounted horizontally 50 cm above the diffuser in a custom enclosure. A spectral scan of this lamp at a fixed high voltage is followed by a spectral scan of the internal 45 W lamp at the same high voltage. This serves to calibrate the internal lamp, and spectral scans of this same lamp at different high voltages determines the responsivity under that operating condition.

### 3.3 Smithsonian Ultraviolet Scanning Radiometer

The Smithsonian SR-18 Ultraviolet Scanning Radiometer measures total solar ultraviolet irradiance at fixed wavelengths selected by 18 interference filters from 290 nm to 324 nm with nominal 2 nm bandwidths. The nominal and actual filter center wavelengths, bandwidths, and maximum filter transmittances of unit UD are given in Table 3.2. The filters are located on a filter wheel, which has a rotational frequency of 15/min underneath a Teflon diffuser. Light from the diffuser passes through each filter in turn, then through a three-aperture collimating apparatus, and is detected by a solar-blind PMT operating in current mode at 23 °C. The output current is converted to voltage and averaged for one minute for each filter. The spectral irradiance responsivity is determined at SERC by operating a calibrated 1000 W FEL-type quartz-halogen lamp in the horizontal position centered 50 cm above the diffuser.

### 3.4 ASRC/RSI Ultraviolet Spectroradiometer

The ASRC/RSI Ultraviolet Spectroradiometer measures total solar ultraviolet irradiance from 280 nm to 400 nm. The instrument consists of a cylindrical housing containing the foreoptics, monochromator, and detector, and a separate box containing the power supply and interface to a remote computer. The cylindrical housing was purged with nitrogen and cooled with recirculating refrigerated water.

The receiving aperture of the foreoptic is a Spectralon disk with a diameter of 2.54 cm and shaped to closely approximate a Lambertian collector. A Spectralon integrating cavity behind the disk collects the transmitted light, and the exit port of this cavity is imaged via a quartz lens onto the entrance slit of the monochromator. A Hg emission lamp placed outside the cavity and near the optic axis is used for wavelength calibration.

The monochromator is a folded double 1/8 m Ebert manufactured by RSI. The diffraction grating is rotated by a sine-drive operated with a stepping motor, and an absolute encoder is linked to the sine-drive. The wavelength of the monochromator in terms of either encoder units or motor steps is determined from spectral scans of the 296.7 nm and 334.1 nm emission lines of the Hg lamp. A linear fit of the centroids of these two lines as a function of encoder units or motor steps yields the wavelength calibration.

The light from the exit slit of the monochromator falls onto a Hamamatsu R2371HA PMT detector maintained at 15 °C by a Peltier cooler. The electrical pulses from the PMT are amplified, discriminated, and divided by five before being transmitted to the counter. The instrument communicates with an external computer over an RS-232 serial line. A microprocessor interprets commands given to the instrument into the appropriate action(s). All spectral scans are performed with increasing wavelength. When the wavelength is decreased, the monochromator moves to a wavelength shorter than the final one, and then the wavelength is increased. The purpose is to remove any mechanical backlash in the monochromator. Additional details on this instrument are given in Ref. [3].

## 4. Atmospheric Conditions

Weather conditions for the Intercomparison were favorable. Prior to the synchronized solar irradiance scans, the only inclement weather was a thunderstorm on the evening of day 168 (sequential day of the year) after all the instruments had been set up on the outdoor pads. During the days of the synchronized scans, the skies were clear in the mornings and increasingly cloudy in the afternoons, especially on day 172.

The temperature, relative humidity, barometric pressure, and wind speed and direction were recorded at the site of the Intercomparison by the instruments listed in Table 1.1. During the days of the synchronized scans (days 169 to 172), the temperature ranged from 10 °C in the early morning to nearly 30 °C in the late afternoon. The relative humidity remained between 20 % and 60 % on each day, while the barometric pressure decreased from 83.3 kPa to 82.8 kPa over the 4 days. The upper air patterns are discussed in Appendix B.

**Fig. 4.1:**
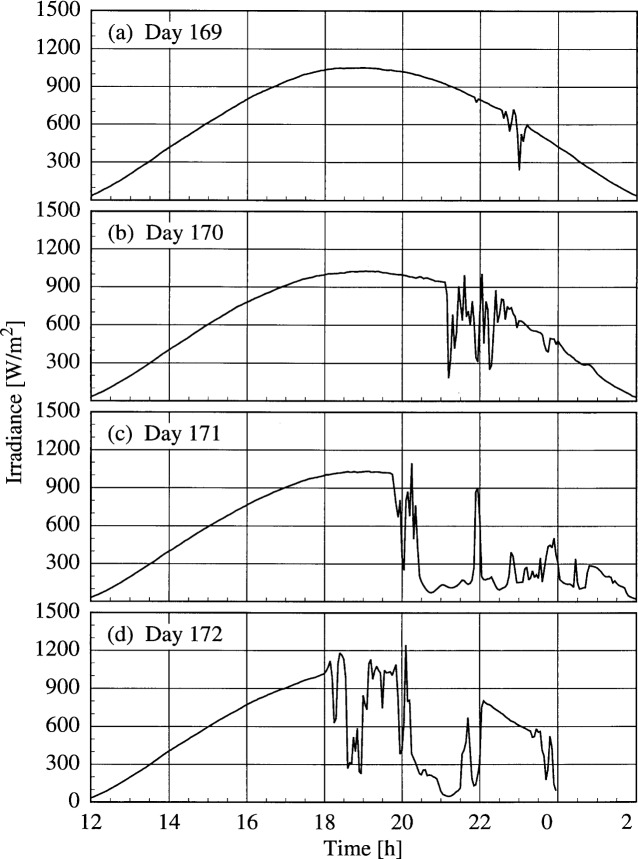
Total horizontal irradiance as a function of time from a solar pyranometer on the days indicated in the panels. Solar noon occurs at approximately 19.0 h UTC.

A set of broadband radiometric instruments, listed in Table 1.1, were located on the test facility platform and made continuous measurements concurrently with the Intercomparison. Results from one solar pyranometer are shown in Fig. 4.1, where the irradiance is plotted as a function of time for each day. This solar pyranometer measured total horizontal irradiance from 280 nm to 3000 nm. The clear morning skies and increasing afternoon cloudiness are evident in Fig. 4.1. On day 169, the sky was clear virtually the entire day. Afternoon cloudiness developed at 21.0 h on day 170 and began earlier on succeeding days (19.5 h and 18.0 h on days 171 and 172, respectively), while the mornings remained clear. Based upon the consistency of the irradiances in the mornings, the turbidity of the atmosphere did not change noticeably during the days of the synchronized scans.

**Fig. 4.2:**
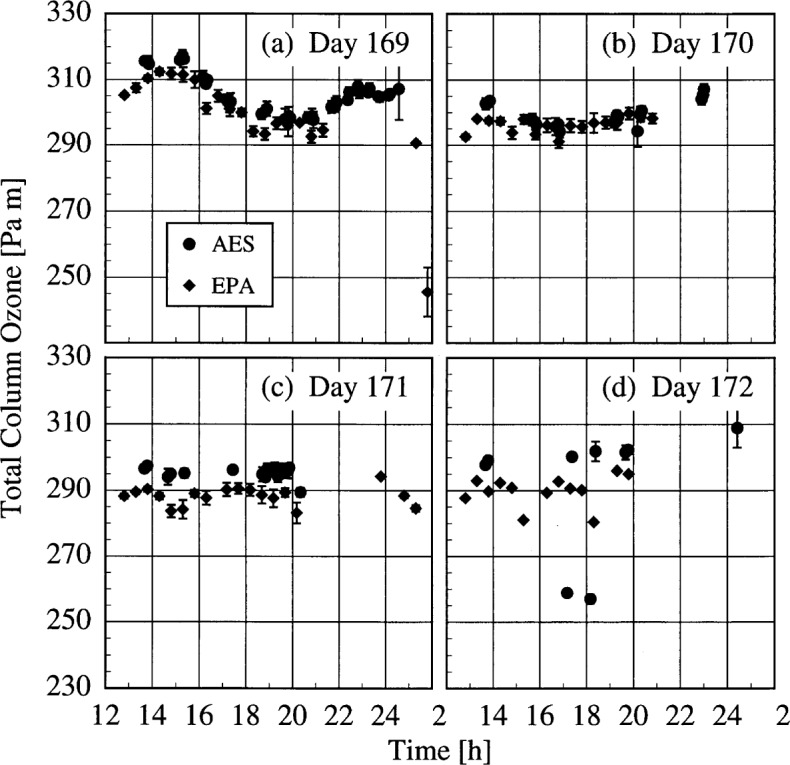
Total column ozone as a function of time on the days indicated in the panels as determined by the instruments indicated in the legend. The vertical bars are the standard deviations of the values.

The AES and EPA instruments determined total column ozone throughout the Intercomparison from measurements of the direct solar beam. The results are shown in Fig. 4.2, where the total column ozone is plotted as a function of time for each day. The vertical bars are the standard deviation of each value. The total column ozone reached a maximum of 310 Pa · m (306 matm · cm) on the morning of day 169, then decreased to between 280 Pa · m and 300 Pa · m (276 matm · cm and 296 matm · cm) for the remainder of the days on which synchronized scans were performed.

## 5. Instrument Characterizations

The spectroradiometers were characterized for the parameters which most affect their ability to accurately measure solar ultraviolet irradiance, and which did not require elaborate experimental equipment or techniques. Therefore, the slit-scattering function, stray-light rejection, wavelength uncertainty, bandwidth, and spectral irradiance responsivity were determined. All of the characterizations were performed both indoors in the concrete building prior to deploying the instruments on the pads and outdoors on the pads. Performing the characterizations both indoors and outdoors allowed the adequacy of the techniques used outdoors to be determined. Since detailed mathematical discussions of the characterization techniques based upon a simple measurement equation have been given previously [2], they will not be repeated here.

### 5.1 Slit-Scattering Function and Stray-Light Rejection

#### 5.1.1 Experimental Procedure

An Omnichrome Model 3056 HeCd laser with a single line at 325.029 nm and a nominal power of 5 mW was used to determine both the slit-scattering function and the stray-light rejection of the instruments. For the characterizations performed indoors, the laser was placed on a table in a darkened room. The output of the laser was directed across the room and reflected off a mirror onto the diffuser of the instrument. The beam diameter was approximately the same diameter as the diffusers. Outdoors, the laser was mounted on a tripod, and a box with a hole was placed on top of the instrument. The output of the laser was directed through the hole directly onto the diffuser. The outdoor measurements were performed at twilight and in the evening to minimize the background signal from the sky.

High-resolution spectral scans were performed near 325 nm to obtain the bandwidth of the instrument, the centroid of the line, and the shape of the slit-scattering function near its peak. Lower-resolution spectral scans were performed across the entire wavelength ranges of the instruments to obtain the full slit-scattering function. For the SERC instrument, the signals were measured for 3 min. The instruments were configured so that the maximum signal did not saturate the PMT. For the AES and EPA instruments, this involved using an internal neutral-density filter for the high-resolution scans, and then removing the filter from the optical path for the low-resolution scans. The PMT high voltage on the NSF instrument was adjusted to prevent the signal from saturating, while the amount of laser light falling on the diffuser of the USDA instrument was adjusted to keep the number of counts below the “roll-over” value of 2^20^. A lower-resolution scan was also performed with the laser beam blocked to check for stray light from sources other than the laser. There were no signals greater than the dark signal either indoors or outdoors for any of the instruments.

#### 5.1.2 Data Analysis

While not important for spectral scans of laser lines because the light is monochromatic, background subtraction is important for spectral scans of lamp emission lines because of the underlying continuous emission from these lamps. To maintain consistency, background subtraction was also performed for spectral scans of laser light. The background signal is described by a linear fit of the signals at wavelengths that differ by 1.5 bandwidths from the wavelength of the peak signal. For unresolved multiple lines in emission lamps, the factor is increased from 1.5 to 2.0. The signals and wavelengths for the first 5 consecutive data pairs that lie outside this range are averaged and fit with a straight line to yield the background signal as a function of wavelength. This fit is subtracted from the signals within the range. There is obviously an interplay between the background subtraction and the bandwidth, but a consistent bandwidth can be obtained after only one or, at most, two iterations between background subtraction and the bandwidth calculation.

The bandwidth of the instrument is defined here as the full-width-at-half-maximum (FWHM) from a high-resolution spectral scan of a laser line or a singlet lamp emission line. Linear interpolation is used to find the wavelengths at which the signal is one-half that of the peak. The bandwidth is then the difference between these two wavelengths.

The centroid method is used as the best estimate of the wavelengths of laser lines and lamp emission lines. The centroid *C* from a high-resolution scan is given by

$$C=\sum_{i}S_{i}\lambda_{i}/\sum_{i}S_{i},$$ (5.1)

where *i* indexes the signals *S* and wavelengths *λ* corresponding to those signals greater than 0.1 times the peak signal.

Because the signal from the laser saturated without a neutral-density filter in the optical path of the EPA instrument, the optical density at 325 nm of a neutral-density filter was determined from the common wavelengths at which signals were measured for scans both with and without the filter. The relation between the signal with the filter, *S*_f_, and the signal at the same wavelength without the filter, *S*_0_, is given by

$$S_{f}=S_{0}\times 10^{-D},$$ (5.2)

where *D* is the optical density. Therefore, the optical density is given by

$$D=\log_{10}(S_{0}/S_{f}).$$ (5.3)

Performing this calculation for the non-saturated signals at each wavelength and averaging the values yields an optical density of 0.97 for the filter. The signal from the laser did not saturate with the AES instrument.

For the high-resolution scans, normalization of the signals by the peak signal was straightforward since there was no saturation of the signal. For the low-resolution scans, however, the normalization is more complicated since the signals from the EPA instrument saturated near 325 nm. Therefore, the peak signal from the high-resolution scan and the optical density of the filter were used in Eq. (5.3) to calculate the peak signal for the scan without the neutral-density filter.

The peak signals obtained in the high-resolution scans were used to normalize the signals from the low-resolution scans for the NSF and USDA instruments since there was no saturation. The peak signal for the SERC instrument was not as readily known since there is no filter centered at 325 nm. Therefore, the peak signal for each filter was obtained from the measured signal of the filter centered at the longest wavelength that did not saturate. These peak signals were calculated by dividing the measured signal from the filter centered at 320.28 nm by the transmittance of that filter at 325 nm and multiplying by the peak transmittance of each filter.

#### 5.1.3 Results and Discussion

The bandwidths of the instruments and the centroids of the laser line are most useful when included with those values obtained from the scans of the Hg and Cd lamps. Therefore, the results from these determinations are shown in Figs. 5.3 and 5.4. The bandwidths at 325 nm are close to the nominal values, 0.6 nm for the AES and EPA instruments, 0.95 nm for the NSF instrument, and 0.3 nm for the USDA instrument.

**Fig. 5.1:**
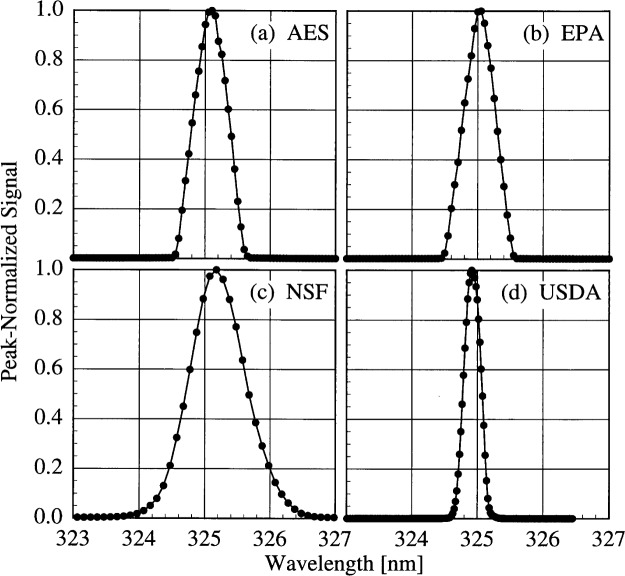
Peak-normalized signal as a function of wavelength from high-resolution spectral scans of the 325.029 nm line from a HeCd laser for the instruments indicated in each panel, demonstrating the slit-scattering functions.

**Fig. 5.2:**
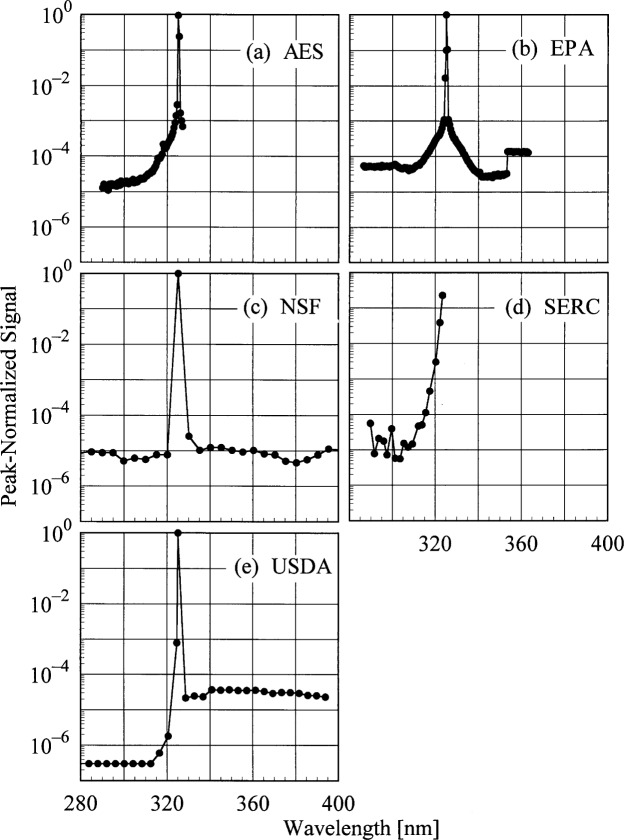
Peak-normalized signal as a function of wavelength from low-resolution spectral scans of the 325.029 nm line from a HeCd laser for the instruments indicated in each panel, demonstrating the stray-light rejections.

The slit-scattering functions are shown in Figs. 5.1 and 5.2, from high- and low-resolution scans, respectively, where the peak-normalized signal is plotted as a function of wavelength. From Fig. 5.1, the slit-scattering functions of the AES, EPA, and USDA instruments are nearly triangular and symmetric about the peak wavelength, while that for the NSF instrument is more Gaussian. The stray-light rejection of each instrument, from Fig. 5.2, is the peak-normalized signal at the shortest wavelengths. The stray-light rejection of approximately 10^−5^ is reasonable for the AES and EPA instruments since they are single-grating instruments. For the NSF instrument, however, the magnitude of the stray-light rejection, 10^−5^, is greater than expected for a double monochromator, probably due to the limited dynamic range possible with the PMT operating in current mode. The USDA instrument, also a double monochromator, has a greater dynamic range and the measured stray-light rejection, nearly 10^−7^, is better than that of the NSF instrument. The increased normalized signal at wavelengths longer than 325 nm is due to fluorescence of the diffuser. The stray-light rejection of the SERC instrument, approximately 10^−5^, is also reasonable for interference-type filters.

### 5.2 Bandwidth and Wavelength Uncertainty

#### 5.2.1 Introduction

Characterizing the instruments in terms of their response to light from Hg and Cd emission line lamps is somewhat more complex than was the case for a HeCd laser both because there is a continuum in addition to the lines and because there can be unresolved multiple lines. However, it is useful because it yields information at several wavelengths about the bandwidth and the wavelength repeatability and uncertainty of the instruments. The wavelength uncertainty is especially important in the UV-B region of the solar spectrum (280 nm to 315 nm) because the irradiance at the Earth’s surface changes rapidly with wavelength, so a small uncertainty in wavelength translates into a large uncertainty in irradiance.

A distinction needs to be made between wavelength calibration and wavelength registration, both of which affect the wavelength uncertainty. The wavelength calibration is the relation between the motor steps that determine the grating angle and the monochromator wavelength, and is determined from the emission lines of a Hg lamp. The wavelength calibration is in general a non-linear function of motor steps. Therefore, the lines from the Cd lamp are especially valuable for determining the wavelength uncertainty since these lines are not used in the original calibrations of the instruments. The wavelength registration is a fixed offset of motor steps from a known position, which is provided by the 302.3 nm line of Hg for the AES and EPA instruments and by the 296.7 nm line for the NSF instrument.

The wavelengths of emission lines from gas lamps are known to a high degree of accuracy; however, the relative intensities of these lines change with lamp and operating condition. Therefore, an Oriel Model 6035 Hg emission lamp was used because of recent measurements of the relative intensities of the lines from this particular model of lamp [4, 5]. The Cd lamp was purchased from BHK, Inc.

#### 5.2.2 Experimental Procedure

The Hg and Cd emission lamps were separately placed horizontally and as close as practical over the diffuser of the instrument. The lamps were warmed up for 10 min and a spectral scan was performed by the instrument. A black cloth was placed over, but not on, the lamps both to reduce background light, especially from the sky, and for safety considerations. The AES, EPA, NSF, and USDA instruments performed spectral scans over their entire operating ranges at 0.05 nm, 0.05 nm, 0.1 nm, and 0.02 nm increments, respectively.

#### 5.2.3 Data Analysis

Background subtraction and calculation of the centroid and bandwidth for each line were performed as detailed in Sec. 5.1.2. Only the bandwidths for single lines were taken to be indicative of the bandwidth of the instrument at that wavelength. The actual centroids of the lines were calculated from the wavelengths and relative intensities of the emission lines for that particular model of Hg lamp and from the published values for Cd emission lines [6].

#### 5.2.4 Results and Discussion

**Fig. 5.3:**
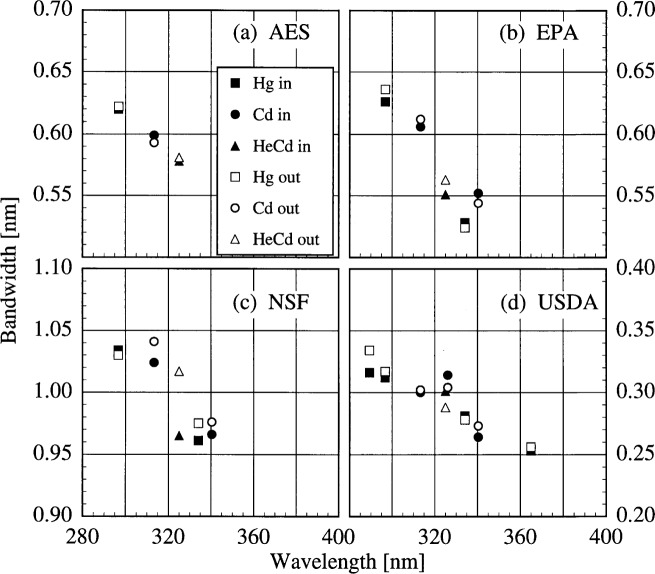
Bandwidth as a function of wavelength for the instruments indicated in each panel from high-resolution spectral scans of the singlet lines from the sources indicated in the legend. The location of the instument, either indoors (in) or outdoors (out), is also indicated in the legend.

**Fig. 5.4:**
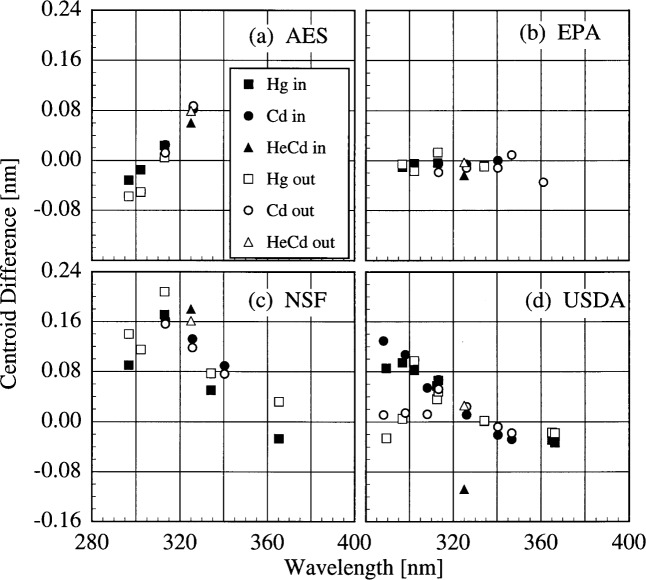
Centroid difference between the calculated and actual values for the instruments indicated in each panel from high-resolution spectral scans of the lines from the sources indicated in the legend, demonstrating the wavelennth uncertainty of each instrument. The location of the instruments, either indoors (in) or outdoors (out), is also indicated in the legend.

The bandwidths calculated from the measurements of singlet Hg and Cd lines and the HeCd line are plotted in Fig. 5.3 as a function of wavelength. Likewise, the differences between the calculated and actual centroids of the Hg, Cd, and HeCd lines are plotted in Fig. 5.4 as a function of wavelength.

The bandwidths of all the instruments decrease with increasing wavelength, and this decrease is consistent between sources and measurements. It is also consistent between instruments, ranging between − 0.2 %/nm and − 0.4 %/nm. Considering only the monochromator, the grating equation predicts that the bandwidth increases with increasing wavelength. Therefore, some other element in the optical path is responsible for the observed decrease. In the detailed discussion below on the NSF instrument, this optical element is identified as the quartz relay lens present in each instrument.

The centroid differences were also consistent between sources and measurements, except at the shortest wavelengths for the USDA instrument. There is a systematic trend for the centroid differences of the AES instrument, with the difference being nearly zero at 302.3 nm, the wavelength at which the monochromator is registered, and as great as 0.08 nm. There is no systematic trend for the EPA instrument, and the differences are nearly all less than 0.02 nm. For the NSF instrument, there is a very significant decrease of the centroid differences, from a maximum of 0.20 nm at the shortest wavelengths to nearly zero at the longest. These results are discussed in more detail below. For the USDA instrument, the discrepancy between the centroid differences measured indoors to those measured outdoors at the shortest wavelengths is likely due to changes in the wavelength calibration. The scans used to calibrate the wavelength in terms of encoder units were both performed outdoors, and so a change in the instrument upon moving outdoors is a likely cause for the discrepancy. Therefore, ignoring this discrepancy, the centroid differences show no systematic trend with wavelength, and the wavelength uncertainty is 0.04 nm.

**Fig. 5.5:**
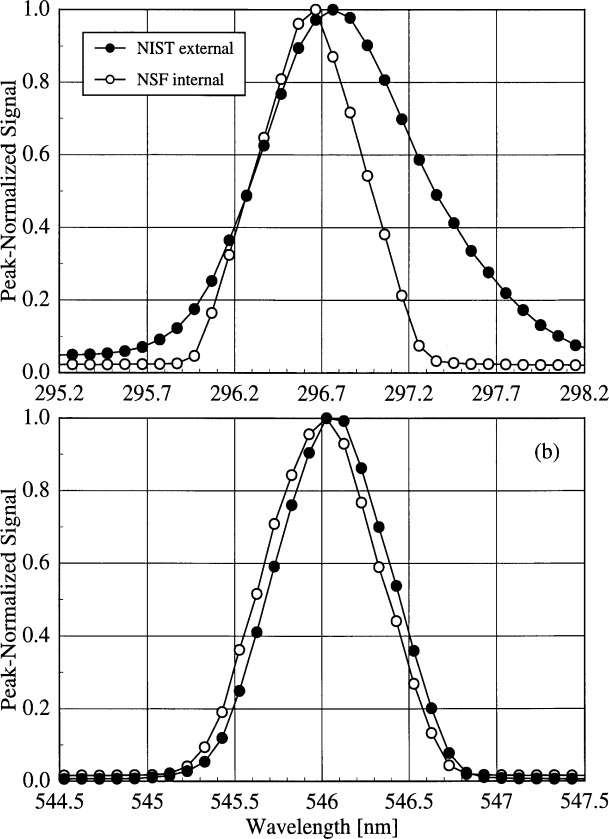
Peak-normalized signal as a function of wavelength for the NSF instrument from high-resolutions spectral scans of the 296.7 nm (a) and 546.1 nm (b) emission lines of the Hg lamps indicated in the legend.

**Fig. 5.6:**
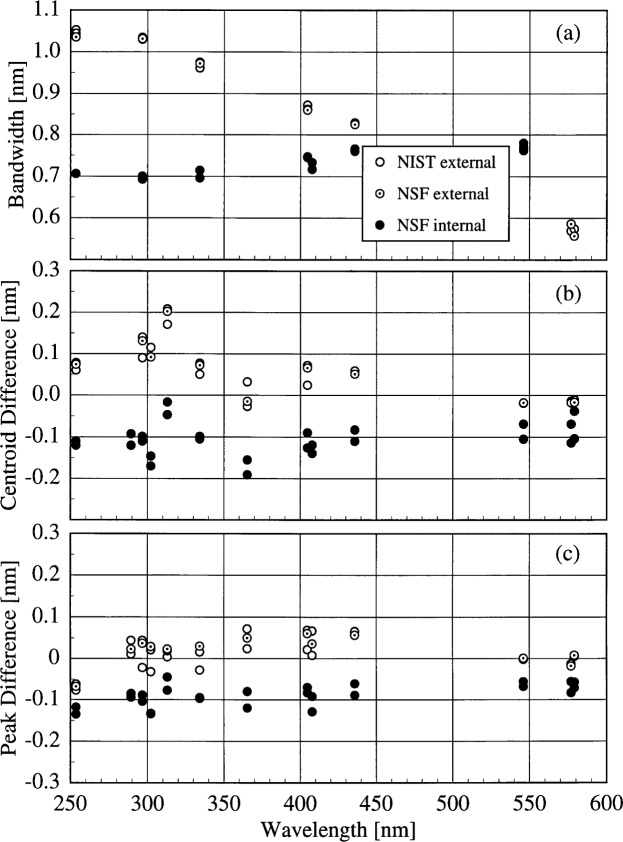
Bandwidth (a), centroid difference (b), and peak difference (c) as a function of wavelength for the NSF instrument from high-resolution spectral scans of the Hg lamps indicated in the legend.

**Fig. 5.7:**
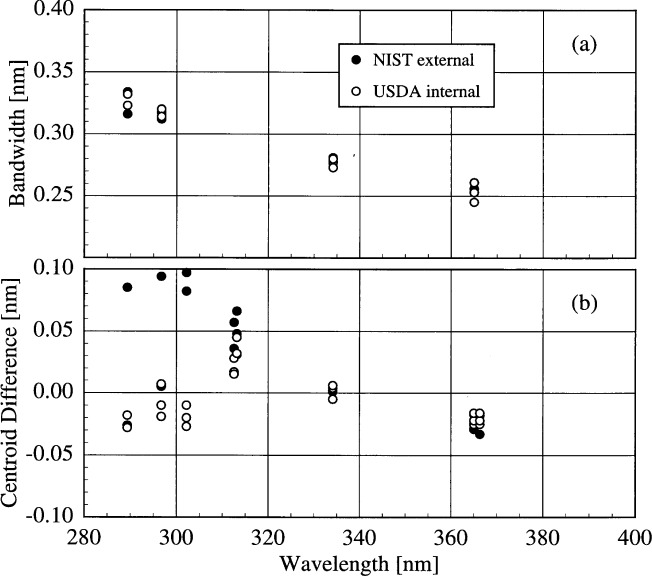
Bandwidth (a) and centroid difference (b) as a function of wavelength for the USDA instrument from high-resolution spectral scans of the Hg lamps indicated in the legend.

The large centroid differences observed for the NSF instrument demand additional explanation. These differences are due both to the methods used to calculate the wavelengths of the lines and to the different optical paths present in the instrument. Plots of the peak-normalized signal as a function of wavelength from spectral scans of the NIST Hg lamp mounted external to the instrument and the internal NSF Hg lamp for two different lines are shown in Fig. 5.5. The line shape at 296.7 nm is much broader for the external lamp than for the internal one, while the line shapes are nearly the same for the two lamps at 546.1 nm. The only difference between the optical paths originating at the diffuser and the internal Hg lamp is a quartz relay lens in the first path. The broader line shape translates into an increased bandwidth, as shown in Fig. 5.6(a), where the bandwidth is plotted as a function of wavelength using results from both the internal and external Hg lamps. The bandwidths calculated from the external lamps decrease with increasing wavelength, while the bandwidths calculated from the internal lamp show the opposite effect and are generally smaller. This increase in bandwidth with increasing wavelength is the trend predicted by the grating equation, and therefore the quartz relay lens is responsible for the decrease observed with the external lamps, not only for the NSF instrument, but for all the instruments shown in Fig. 5.3. This is further corroborated by results from the USDA instrument, which also has an internal Hg lamp, but whose optical path is the same for this lamp and the diffuser. The bandwidth as a function of wavelength is shown in Fig. 5.7(a), and is consistent for both lamps. The bandwidths obtained with the external lamps are appropriate for characterizing the complete instrument performance since the optical path from diffuser to detector is used when measuring solar ultraviolet irradiance. Because of the different line shapes obtained for the NSF instrument, the normal wavelength calibration of this instrument using the internal Hg lamp was corrected using the results obtained with the external Hg lamp.

The method used to calculate the wavelength from the spectral scan of an emission line can have a pronounced effect on the calculated wavelength uncertainty. For the NSF instrument, the wavelength of a line is taken to be that of the peak signal. However, this method is not the most appropriate. Instead, as detailed in Appendix C, the centroid of the measured signals is more appropriate, based upon an analysis using the measurement equation. For signals that are symmetric in wavelength about the peak signal, line wavelengths obtained using the two methods should agree. However, for asymmetric signals, the two methods can yield line wavelengths that are significantly different. This is illustrated in Fig. 5.5(a), where the wavelengths of the peak signals for the two scans differ by only 0.1 nm, while the wavelengths of the centroids differ by nearly 0.2 nm. The centroid differences of the NSF instrument calculated for the internal and external Hg lamps are shown in Fig. 5.6(b) as a function of wavelength. The centroid differences are offset by about − 0.1 nm for the internal lamp with respect to the peak wavelengths, whereas the centroid differences for the external lamps are as great as 0.2 nm and are consistent between lamps. The discrepancy between the centroid differences determined from the internal and external lamps decreases with increasing wavelength since the line shapes become more similar with increasing wavelength. In contrast, the peak differences of the NSF instrument are shown in Fig. 5.6(c) as a function of wavelength. These differences are within ± 0.1 nm for the external lamps and are again offset by about − 0.1 nm for the internal lamp. For the USDA instrument, the centroid difference as a function of wavelength is consistent between the internal and external lamps, as shown in Fig. 5.7(b), since the optical paths are nearly the same. Because the method for calculating the wavelength from a spectral scan of an emission line can have a dramatic effect on the corresponding wavelength calibration, a common method should be used by all ultraviolet monitoring instruments.

### 5.3 Spectral Irradiance Responsivity

#### 5.3.1 Introduction

Measuring the spectral irradiance responsivity (hereafter termed simply the responsivity) of the instruments, both with the NIST standard lamps and with the standard lamps of the participants, was the most important characterization performed at the intercomparison. As at the 1994 Intercomparison, these measurements determined the agreement between the spectral irradiance scales, the translational and temporal stability of the instruments, and the responsivity of each instrument for the synchronized solar irradiance measurements. In addition, the operation of the new NIST field calibration unit was tested.

The responsivity of every instrument was determined indoors in the concrete building and outdoors on the pads. The NIST standard lamps were operated both on a tripod and in the field calibration unit for the indoor measurements, and only in the field calibration unit for the outdoor measurements. The participants used their own lamp enclosures both indoors and outdoors.

#### 5.3.2 Experimental Procedure

The field calibration unit was developed to avoid the difficulties encountered at the 1994 Intercomparison when using the tripod outdoors for responsivity measurements. Alignment of the lamp over the diffuser was a time-consuming process that had to be repeated with each measurement on each instrument, and the scans had to be performed at night with complete darkness since the lamp was open to the surroundings. The field calibration unit consists of three circular baffles, 45 cm in diameter and separated by 15 cm, with a mount for a horizontal lamp on the top baffle. A light trap above the lamp and shrouding around the baffles enclose the lamp, isolating it from the surroundings, and the unit mounts on an instrument interface plate, which is the key to the utility of the field calibration unit. Each instrument has an interface plate specifically designed to fit around the diffuser and rest on top of the instrument. The interface plate also sets the distance from the diffuser to the lamp at 50.0 cm by using spacers machined to the appropriate height. Additional details of the field calibration unit are given elsewhere [8].

The lamp mount on the field calibration unit was adjusted once to center the lamp 50.0 cm above the diffuser. The unit was placed on the SERC instrument and a rod attached to a cup, both made from Al, were placed over the diffuser. The rod and cup were machined so that the top of the rod was 50.0 cm above the diffuser. A lamp alignment jig was placed in the lamp mount and the mount was adjusted so that the jig was centered on and touching the rod. The mount was then locked in place and the distance from the diffuser to the jig was checked with the stick used to set the distance with the tripod system. The location of the mount was then checked on each instrument, and no further adjustments were necessary. Therefore, for each responsivity measurement using the field calibration unit, all that was required for alignment was to place the appropriate interface plate on the instrument and mount the unit on the interface plate.

The spectral irradiance of the 1000 W FEL-type NIST standard lamps, designated OS-27 and F-332, had been determined in the horizontal position using a method similar to the one described in Ref. [7]. The responsivity of each instrument was determined indoors with lamp OS-27 mounted on the tripod using the same technique as at the 1994 Intercomparison [2]. The responsivity was then determined indoors with lamp F-332 mounted in the field calibration unit using the same power supply as with lamp OS-27. This was done to establish the equivalence between the responsivities obtained with the tripod and with the field calibration unit since this was the first time the unit had been used. The responsivity of each instrument was determined at least twice outdoors with lamp F-332 mounted in the field calibration unit.

For all determinations of responsivity using a NIST lamp, spectral scans were performed with a 3.5 cm wide shutter located halfway between the lamp and the diffuser to measure the diffuse signal, and without the shutter to measure the total signal. For both Brewer instruments, the wavelength registration was set prior to measuring the responsivity. Spectral scans were performed from 290 nm to 325 nm and from 286.5 nm to 360 nm for the AES and EPA instruments, respectively. All scans were at a 3.5 nm increment, and were with both increasing and decreasing wavelength for the AES instrument and only for increasing wavelength for the EPA instrument. Two scans were performed for the diffuse signal and two scans for the total signal. Spectral scans with the NSF instrument were from 275 nm to 330 nm with a PMT voltage of 995 V and from 275 nm to 410 nm with a voltage of 865 V, all with a 1.0 nm increment and increasing wavelength. Two scans were performed for the diffuse signal and two scans for the total signal. In addition, scans were performed with the internal shutter closed to measure the dark signal. Both the diffuse and total signals from the SERC instrument were collected for ten minutes. The USDA instrument performed spectral scans from 280 nm to 400 nm at a 0.2 nm increment with increasing wavelength, one scan for the diffuse signal and one scan for the total signal.

There were two problems associated with using the field calibration unit. One was that the interface plate for the AES instrument did not fit correctly—the viewing window was larger than the size for which the interface plate was designed. Therefore, the interface plate was placed on blocks on top of the instrument, and different spacers were used to set the distance from the diffuser to the lamp at 50.0 cm. Because of problems with the AES instrument described below, its responsivity was determined twice indoors with the field calibration unit. Unfortunately, the distance from the diffuser to the lamp was mistakenly set incorrectly both times, first 0.16 cm (1/16 in) too high and then 0.16 cm (1/16 in) too low. This was corrected for the outdoor measurements.

The other problem was discovered after the first day of measurements with the field calibration unit indoors. The responsivities obtained with lamp OS-27 on the tripod mount did not agree with those obtained with lamp F-332 in the field calibration unit. To determine the reason for the discrepancy, the responsivity of the SERC instrument was measured using both lamps on the tripod mount. The same discrepancy was obtained, implying that there had been a change in the spectral irradiance of one of the lamps from the time it was calibrated at NIST to the time when it was used at the Intercomparison. Therefore, the lamps were checked periodically throughout the Intercomparison by bringing the SERC instrument indoors and determining its responsivity using both lamps, OS-27 on the tripod mount and F-332 in the field calibration unit. The discrepancy remained consistent throughout the Intercomparison, and upon returning to NIST it was determined that the spectral irradiance of lamp F-332 had changed before the Intercomparison but had remained stable after that time.

The participants with the AES instrument used 1000 W DXW-type quartz-halogen lamps mounted 40 cm above the diffuser in a custom enclosure. The lamps were designated S-702, S-789, and U-5. The EPA instrument used 50 W quartz-halogen lamps, mounted 5 cm above the diffuser in an enclosure, supplied and calibrated by Sci-Tec, Inc., and designated 326, 327, 328, 329, and 330. The NSF instrument used 200 W DXW-type quartz-halogen lamps, supplied and calibrated by Optronics Laboratories, mounted 50 cm above the diffuser in a custom enclosure. The lamps were designated M-761 and M-767. All the enclosures allowed the participants to determine the responsivity in the normal orientation of the instrument both indoors and outdoors. The responsivity of the SERC instrument had been determined at the home laboratory with a 1000 W FEL-type quartz-halogen lamp, supplied and calibrated by Eppley Laboratories, designated EN-73.

A schedule of the spectral scans of standard lamps is given in Table 5.1, along with the corresponding instrument temperatures. The spectral scans on days 165 to 167 were performed indoors, while the remainder were outdoors. The exceptions are the scans on day 170 and the last two scans on day 173 for the SERC instrument, which were performed indoors to check the lamps, as described above.

#### 5.3.3 Data Analysis

From spectral scans of a standard lamp, the responsivity is given by dividing the signal by the lamp irradiance. For the NIST standard lamps, the signal was the direct signal, given by the difference between the total signal and the diffuse signal. However, for the participants’ lamps, the signal was the total signal since a shutter was not used to measure the diffuse signal. The spectral irradiances of the standard lamps were fit with a cubic spline interpolation to the wavelengths of the signals. The NIST standard lamps had been calibrated from 275 nm to 405 nm. To cover the wavelength range measured by the instruments, the calibration was extended to 265 nm and 415 nm by fitting the spectral irradiances with the Wien approximation of the Planck equation, multiplied by a second-order polynomial.

For the NSF instrument, the dark signals were averaged and subtracted from the average signal at each wavelength with the internal shutter open. For the AES, EPA, NSF, and SERC instruments, the signals at each wavelength from multiple scans were averaged. The standard uncertainties in the signals were the standard deviations of the mean, and these were propagated through to the direct signals. Since multiple scans were not performed with the USDA instrument, the standard uncertainties of the signals, from Poisson statistics, were the square roots of the signals. These standard uncertainties were propagated through the calculation of the final responsivity, which consisted of smoothing the responsivity twice with a five-point triangle and then linearly interpolating to integral wavelengths at a 1 nm increment.

The uncertainty analysis for the responsivities is similar to the approach given in Refs. [2] and [7], and the details are presented in Appendix D. Components of uncertainty arise from the standard lamp (spectral irradiance, size of diffuser, goniometric distribution, and current), the alignment of the lamp, and the instrument (wavelength and signal). The relative standard uncertainties arising from each component are given in Table 5.2 at selected wavelengths for the first outdoor determination of responsivity with the field calibration unit. The relative standard uncertainties are combined in quadrature for both random and systematic effects. The greatest systematic component is the irradiance of the standard lamp, while the greatest random component is the signal. Note that these uncertainties apply only to the NIST standard lamps. For the participants’ lamps, only the uncertainties arising from the instrument (the wavelength and signal) are known.

The separation of uncertainties between random and systematic effects is important when comparing responsivities. For example, the relative standard uncertainty in the relative difference between the responsivities determined by a NIST standard lamp and by a participant’s lamp includes components of uncertainty arising from both random and systematic effects. However, the relative standard uncertainty in the relative difference between two responsivities determined by a NIST standard lamp includes components of uncertainty arising only from random effects.

#### 5.3.4 Results and Discussion

The spectral irradiance of each participant’s lamp is based upon the spectral irradiance scale used by that participant’s monitoring network. These scales, in turn, are based upon calibrated lamps supplied by different manufacturers. A comparison between these scales and the NIST spectral irradiance scale is very important to assess the accuracy of the participants’ scales. The relative difference between a participant’s spectral irradiance scale and the NIST scale is given by the relative difference between the responsivity using the NIST standard lamp and the responsivity using the participant’s standard lamp. Note that the relative difference between two values *x* and *y* is given by (*x* − *y*)/*y* = *x*/*y* − 1.

**Fig. 5.8:**
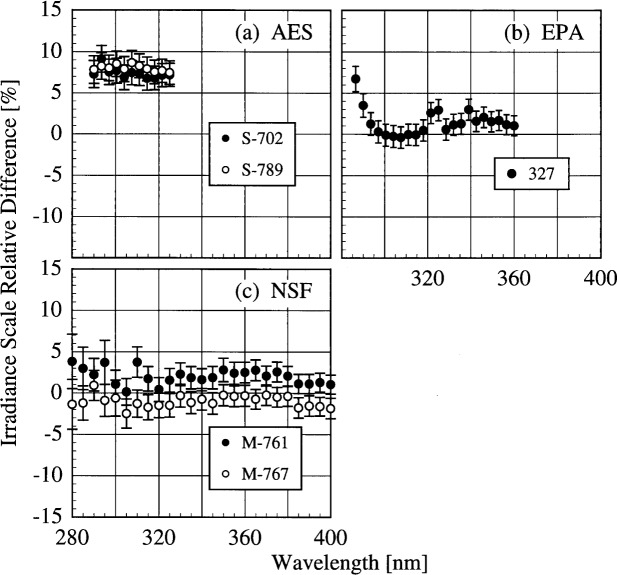
Relative difference between the participants’ spectral irradiance scales and the NIST spectral irradiance scale as a function of wavelength from spectral scans performed indoors. The instruments are indicated in each panel, the participant’s lamps are indicated in the legends, and the vertical lines are the standard uncertainties.

**Fig. 5.9:**
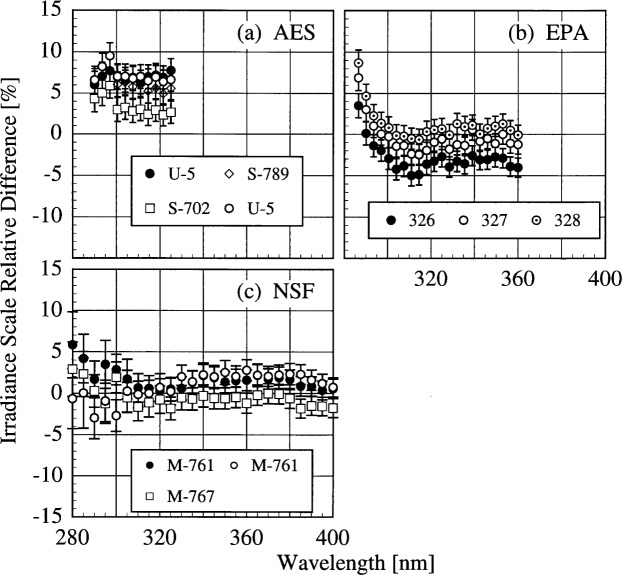
Relative difference between the participants’ spectral irradiance scales and the NIST spectral irradiance scale as a function of wavelength from spectral scans performed outdoors. The instruments are indicated in each panel, the participant’s lamps are indicated in the legends, and the vertical lines are the standard uncertainties.

**Fig. 5.10:**
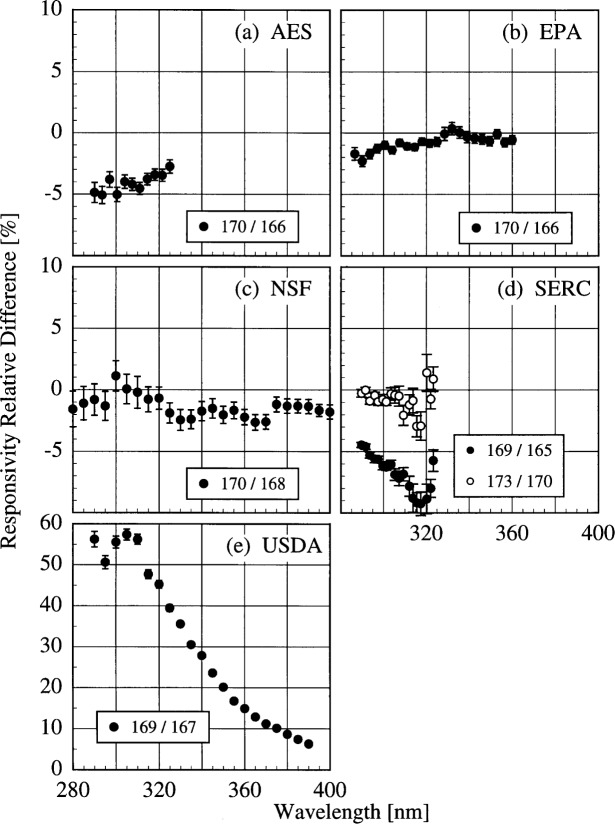
Relative difference using the NIST standard lamp between the responsivity determined outdoors and the responsivity determined indoors as a function of wavelength, indicating the translational stability of the instruments. The instruments are indicated in each panel, the days on which the responsivities were determined are indicated in the legends, and the vertical lines are the standard uncertainties.

The participant responsivities used for this comparison were those that were determined on the same day as a scan of the NIST standard lamp. The relative difference between the participant’s spectral irradiance scale and the NIST scale as a function of wavelength is shown in Fig. 5.8 for scans performed indoors and in Fig 5.9 for scans performed outdoors. The vertical bars are the combined standard uncertainties of the differences using components arising from both random and systematic effects. The lamps, times, and instrument temperature changes used for the differences in these two figures, as well as in Figs. 5.10 to 5.13, are listed in Table 5.3.

The spectral irradiance scales from the lamps used by AES are consistently greater than the NIST scale by as much as 10 % both indoors and outdoors. The scales from the lamps used by EPA and NSF are within ± 5 % of the NIST scale both indoors and outdoors. There is no dependence on wavelength for the NSF scale, while the relative difference between the EPA scale and the NIST scale increases rapidly for wavelengths shorter than 300 nm. There is therefore a systematic error with the EPA scale at the shortest wavelengths. This comparison of the spectral irradiance scales used by the participants indicates agreement within − 5 % to + 10 % of the NIST scale.

The responsivity of every instrument was determined both indoors and outdoors. Therefore, the difference between the responsivity determined outdoors with a particular lamp and the responsivity determined indoors with the same lamp indicates the stability of the instrument to movement, termed translational stability.

**Fig. 5.11:**
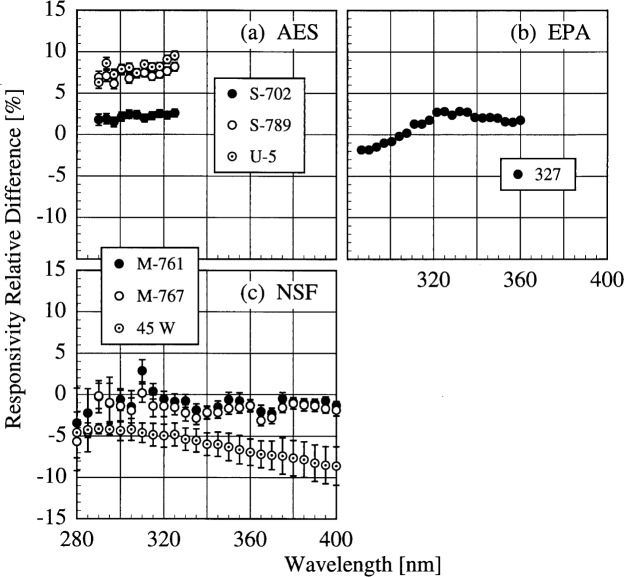
Relative difference using the participants’ lamps between the responsivity determined outdoors and the responsivity determined indoors as a function of wavelength, indicating the translational stability of the instruments. The instruments are indicated in each panel, the participant’s lamps are indicated in the legends, and the vertical lines are the standard uncertainties.

The translational stability was determined using both the NIST standard lamp and participants’ lamps. The first outdoor measurement of responsivity with a lamp that had also been scanned indoors was used for the determination. The relative difference between the responsivity determined outdoors and that determined indoors as a function of wavelength is shown in Fig. 5.10 from spectral scans using the NIST standard lamp and in Fig. 5.11 from spectral scans using the participants’ lamps. The vertical bars are the combined standard uncertainties of the differences using components arising from only random effects.

The responsivity of the AES instrument decreased by approximately 5 % according to the NIST lamp, while it increased by between 2 % and 10 % according to the participant’s lamps. As discussed below, this discrepancy is likely caused by a problem with the PMT voltage. The responsivity of the EPA instrument was stable to within − 2 % to 0 % with the NIST lamp, and to within − 2 % to + 3 % with the participant’s lamp. The decrease in the difference with decreasing wavelength for wavelengths shorter than 325 nm in Fig. 5.11(b) is likely caused by the increased instrument temperature between the two responsivity measurements, as shown in Table 5.3 and detailed in Ref. [2]. The responsivity of the NSF instrument was stable to within ± 3 % from both the NIST lamp and the participant’s external lamps, with no discernible wavelength dependence. However, according to the internal lamp, the responsivity decreased by − 4 % to − 8 % with a definite wavelength dependence. Since this change is not observed with the external lamps, the results indicate that the internal lamp had changed rather than the instrument. Further results with the internal lamp are given below. The SERC instrument responsivity changed by − 4 % to − 10 % upon first moving outdoors. The stability improved upon the second movement, with the responsivity changing by only − 4 % to + 2 %. Finally, the responsivity of the USDA instrument changed by as much as 60 % with a severe wavelength dependence. This instability is discussed in greater detail in Ref. [3].

The responsivity of every instrument was determined twice outdoors using the NIST standard lamp, and most participants also determined responsivities several times outdoors based upon their lamps. The difference between responsivities determined with the same lamp at two different times indicates the temporal stability of the instrument.

**Fig. 5.12:**
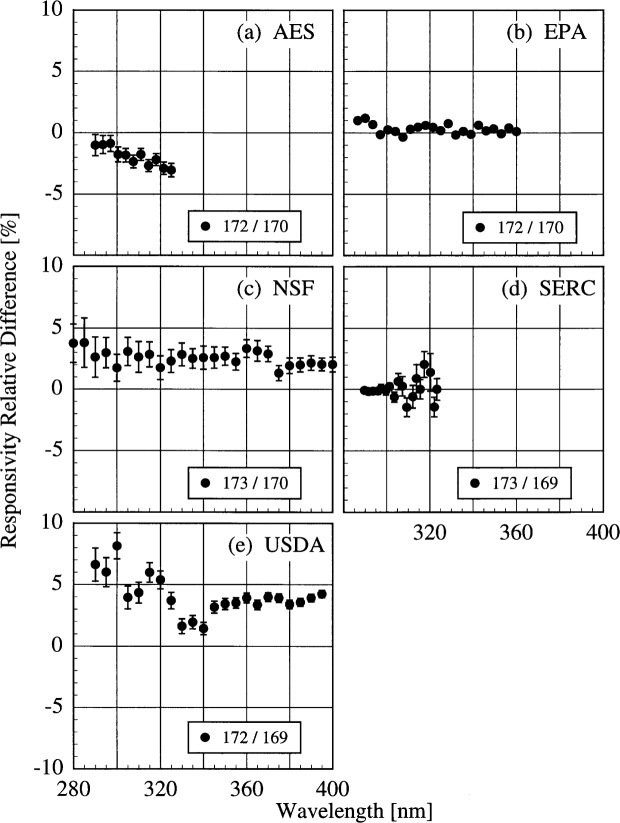
Relative difference using the NIST standard lamp between two responsivities determined outdoors as a function of wavelength, indicating the temporal stability of the instruments. The instruments are indicated in each panel, the days on which the responsivities were determined are indicated in the legends, and the vertical lines are the standard uncertainties.

**Fig. 5.13:**
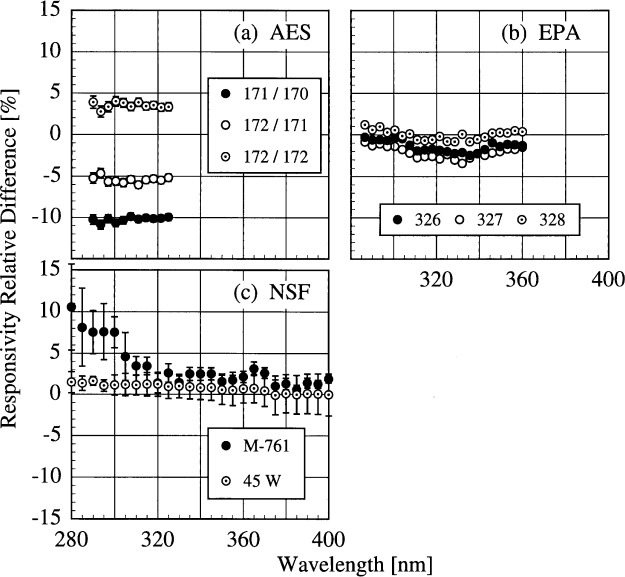
Relative difference using the participants’ lamps between two responsivities determined outdoors as a function of wavelength, indicating the temporal stability of the instruments. The instruments are indicated in each panel, the days (a) on which the responsivities were determined using lamp U-5 and participants’ lamps (b) and (c) are indicated in the legends, and the vertical lines are the standard uncertainties.

The relative difference between the responsivity determined outdoors and a previous determination with the same lamp outdoors as a function of wavelength is shown in Fig. 5.12 from spectral scans using the NIST standard lamp and in Fig. 5.13 from spectral scans using the participant’s lamps. The vertical bars are the combined standard uncertainties of the differences using components arising from only random effects.

The results with the NIST standard lamp indicate that the responsivities of the instruments, with the exception of the USDA instrument, are stable to within several percent over several days with no wavelength dependence. In the best case, the EPA instrument was stable to within ± 1%. While the temporal stability of the USDA instrument was much better than its translational stability, the responsivity still changed by as much as + 9 %. The temporal stability determined by the participants’ lamps is less impressive. The relatively large changes in responsivity for the AES instrument were caused by an instability of the PMT voltage that was not corrected until 16.0 h on day 171. Therefore, the first two differences shown in Fig. 5.13(a) indicate the instability associated with the PMT voltage, while the third difference indicates that the instrument was stable to within + 4 %. According to the participant’s lamps, the EPA instrument was stable to within − 4 % to + 1 %. Paradoxically when compared with Fig. 5.11(c), the stability of the NSF instrument determined with the external lamp was worse than the internal lamp.

**Fig. 5.14:**
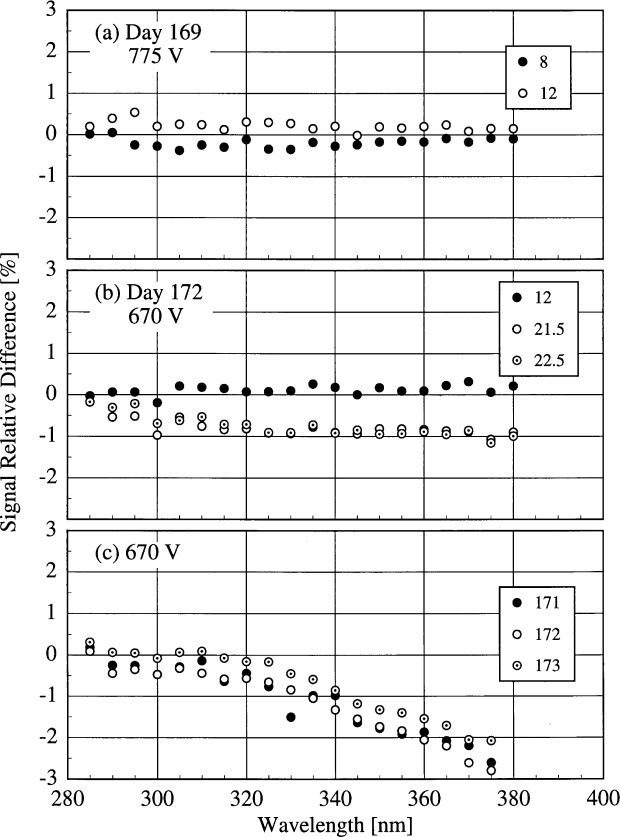
Relative difference between signals measured from the internal 45 W lamp of the NSF instrument at different times and PMT voltages (indicated in each panel) demonstrating the temporal stability of this lamp. In (a) and (b) the initial times are 5 h and 8 h, respectively, and the succeeding times are given in the legends; in (c) the initial day is 169 and the succeeding days are given in the legend.

The signal from the internal 45 W lamp of the NSF instrument was measured repeatedly throughout the Intercomparison. The temporal stability of this lamp is given by the relative difference between signals obtained at the same PMT voltages at different times. This is shown in Fig. 5.14, where the relative difference is plotted as a function of wavelength. On the same day, as shown in Figs. 5.14(a) and 5.14(b), the internal lamp is stable to within ± 1 %. From Fig. 5.14(c), the output of the lamp decreased by up to 3 % from day 169 to 171, but again remained stable to within ± 1 % on succeeding days.

The conclusions to be drawn from the determinations of responsivity are similar to those from the 1994 Intercomparison. The discrepancies between the participants’ spectral irradiance scales and the NIST scale were between − 10 % and + 10 % in 1994 and − 5 % and + 10 % in 1995. This illustrates the need to use a set of common standard lamps within a network and between networks so that the irradiance scales for all instruments are the same. The responsivities changed upon movement of the instruments, and this was especially pronounced for the USDA instrument. Therefore, the responsivity of an instrument must be determined at the monitoring site. The responsivity changes outdoors were within ± 5 %, as they were in 1994, indicating relatively good temporal stability. Unlike the results in 1994, the temperature changes between determinations of responsivity for the AES and EPA instruments were not sufficient to illustrate the effect of temperature on responsivity.

**Fig. 5.15:**
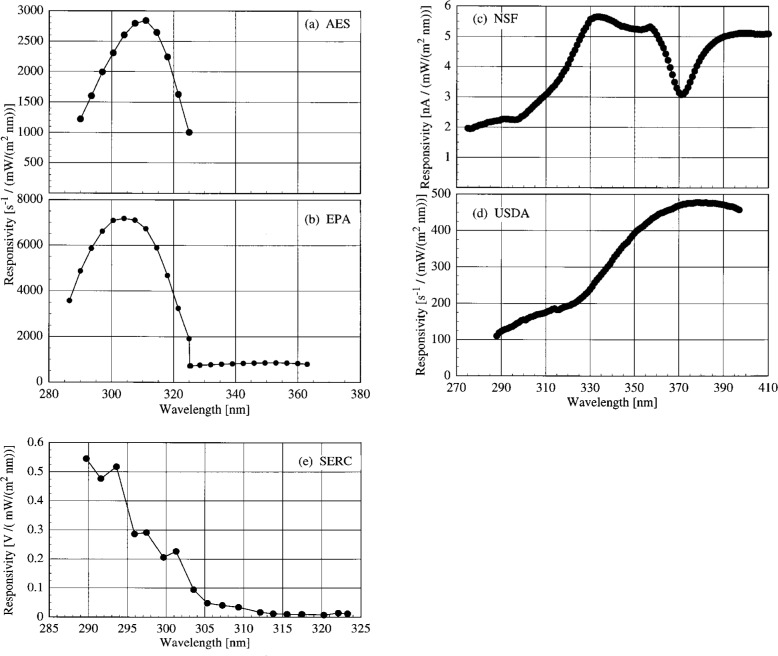
Responsivity as a function of wavelength for each instrument indicated in the panels.

The responsivities determined outdoors using the NIST standard lamp were used to calculate the irradiances the synchronized solar scans. Using a common standard for responsivity simplifies intercomparisons between measured irradiances since differences between spectral irradiance scales are removed from the analysis. Therefore, actual instrument performances can be evaluated more readily. The responsivities of the instruments as a function of wavelength are shown in Fig. 5.15 for (a) the AES instrument, (b) the EPA instruments, (c) the NSF instrument, (d) the USDA instrument, and (e) the SERC instrument. The peaks in the responsivities of the AES and EPA instruments between 300 nm and 320 nm are due to the NiSO_4_ filters, while the responsivities of the NSF and USDA instruments are dominated by the fore-optics and monochromators, and the responsivity of the SERC instrument is dominated by the PMT.

## 6. Solar Irradiance

### 6.1 Introduction

The ultimate goal of the Intercomparison was to have all the instruments measure the solar ultraviolet irradiance concurrently, which was achieved over several days of the Intercomparison. The solar ultraviolet irradiance *E*(*λ*_0_) was calculated from the measured signals *S*(*λ*_0_) using the simplified measurement equation

$$E(\lambda_{0})=S(\lambda_{0})/R(\lambda_{0}),$$ (6.1)

with the responsivity *R*(*λ*_0_) for each instrument being that determined from outdoor scans of the NIST standard lamp. This was done to provide a common irradiance scale for all the instruments, thereby removing discrepancies caused by different scales and facilitating comparisons between instruments.

### 6.2 Experimental Procedure

Synchronized spectral scans of the solar ultraviolet irradiance began on the hour and half-hour from wavelengths of 290 nm to 325 nm at increments of 0.25 nm with 3 s between each wavelength. This range was common to all the instruments, and the EPA and NSF instruments continued scanning to longer wavelengths. The participants used their own scan routines on day 171, again beginning on the hour and half-hour. The purpose was to obtain irradiances using the normal operating procedures of the instruments, to determine if these procedures had any effect on the intercomparability of the instruments. The clock for each instrument was set daily from a common clock synchronized with the satellite Global Positioning System. The synchronized scans lasted 7 min, except on day 171, and the maximum discrepancy in time between instruments during these scans was 2 s. Other measurements, such as wavelength calibrations and total column ozone, were performed by some instruments during the times between synchronized scans.

The days, times, and participating instruments for the synchronized solar scans used in the analyses below are listed in Table 6.1. The AES instrument was not operating correctly until after 16.0 h on day 171 due to problems with the high voltage on the PMT. Likewise, the USDA instrument was not operating for most of day 171 because corrections were being made to the wavelength drive.

### 6.3 Data Analysis

For all instruments, the measured signal was corrected before the irradiance was calculated. For the AES and EPA instruments, the signal was converted to a photon rate as detailed in Sec. 3.1 with dark subtraction and dead-time correction. The wavelengths of the NSF instrument were corrected as detailed in Sec. 3.2, while dark subtraction was performed by averaging all the signals at wavelengths shorter than 290 nm and subtracting this value from all the signals of the scan. Dark subtraction and averaging the signals over the 7 min of the synchronized scans was performed for the SERC instrument. The average of the dark signals obtained immediately before and after a synchronized scan of the USDA instrument was subtracted from all the signals of the scan.

The stray-light rejections of the instruments, shown in Fig. 5.2, can result in relatively large signals at the shortest wavelengths. To account for this, stray-light subtraction was employed for the AES and EPA instruments. The signals at wavelengths shorter than 292 nm were averaged and subtracted from all signals from the scan. It was these signals with the stray-light subtraction that were divided by the responsivity to obtain the solar ultraviolet irradiance. The subtraction used for the NSF instrument is also a stray-light subtraction, although the signals obtained in a darkened room with no source illuminating the diffuser are the same as those obtained with the solar spectral scans at wavelengths shorter than 290 nm. Therefore, the contribution to the signal due to stray-light is indistinguishable from the dark signal. The stray-light rejection of the USDA instrument was sufficiently large that no correction to the signals at the shortest wavelengths was necessary.

The method used to determine the responsivity of the NSF instrument during solar scans complicated the data analysis. The usual procedure with this instrument is to transfer the spectral irradiance scale of the external 200 W lamp to the internal 45 W lamp from spectral scans of both lamps with the same high voltage on the PMT. Different high voltages are used for scans of the solar irradiance, and the responsivity of the instrument is dependent upon the high voltage. Therefore, the internal lamp is scanned at least daily at these high voltages to determine the responsivity under these conditions. To use the NIST irradiance scale with this procedure, the scale was transferred to the NSF external 200 W lamp from the first outdoor scan of the NIST standard lamp, and this new scale for the external lamp was then used with scans of the internal 45 W lamp. The responsivity of the instrument at any high voltage was determined from the scan of the 45 W lamp at the same high voltage that occurred closest in time to the scan of the solar irradiance.

To maintain consistency with the NSF procedure for responsivity, and because the responsivity of the USDA instrument drifted over time, the responsivities used to calculate the solar irradiance were those determined closest in time to the synchronized scans. Using this procedure, the relative standard uncertainty in responsivity due to drifts is estimated to be 1 % for all instruments. The days and times of the responsivities used for the solar irradiances are given in Table 6.2. The responsivities of the EPA instrument were extrapolated to 325.25 nm and 363 nm using second-order polynomial fits. From Eq. (6.1), the irradiance at a given wavelength is the signal at that wavelength divided by the responsivity at that same wavelength. Because the responsivities were not determined at all the wavelengths of the synchronized solar scans, the responsivities at these wavelengths were calculated from natural cubic spline interpolations.

### 6.4 Results and Discussion

**Fig. 6.1:**
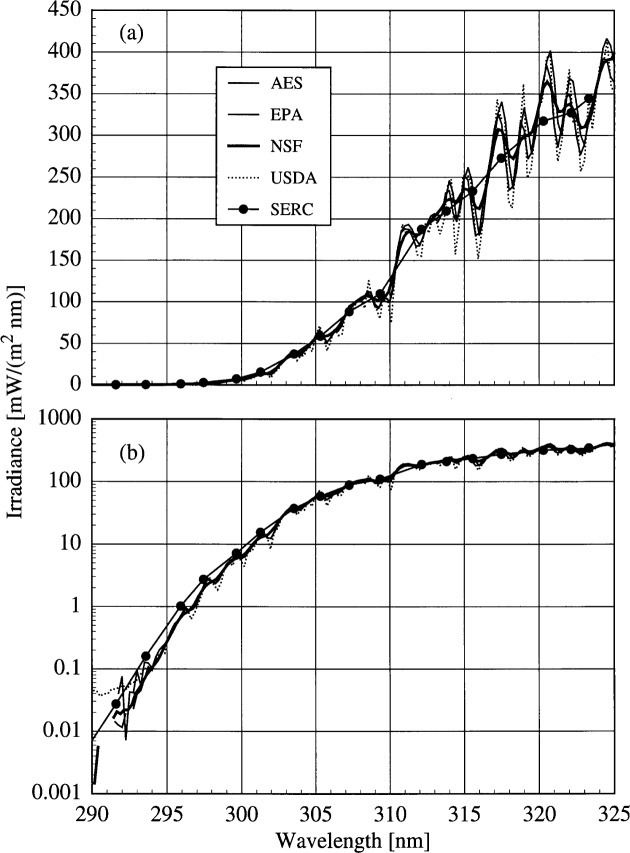
Solar irradiance on a linear scale (a) and on a logarithmic scale (b) as a function of wavelength determined by the instruments indicated in the legend on day 172 at 17.0 h.

The solar irradiance as a function of wavelength determined by all instruments from a synchronized spectral scan on day 172 at 17.0 h is shown in Fig. 6.1. The irradiance is plotted on a linear scale in Fig. 6.1(a) and on a logarithmic scale in Fig. 6.1(b). This figure illustrates the challenges encountered in accurately measuring the solar ultraviolet irradiance, especially in the UV-B wavelength region, and of comparing the results between instruments. The outstanding feature of ground-level solar ultraviolet irradiance is its rapid decrease with decreasing wavelength in the UV-B region due to absorption by ozone, as illustrated in Fig. 6.1(b). The irradiance decreases by nearly five orders of magnitude from 325 nm to 290 nm, which imposes stringent requirements on the instruments in terms of wavelength accuracy and stray-light rejection. In the region of steepest decrease, a relatively small uncertainty in wavelength translates into a relatively large uncertainty in irradiance. An accurate measurement of the irradiance at the shortest wavelengths requires the best possible stray-light rejection so the signal is not dominated by light from wavelengths longer than the nominal one.

The moderately structured nature of the solar spectral irradiance, as shown in Fig. 6.1(a) for wavelengths greater than 310 nm, complicates comparisons between instruments. While the structure of the spectral irradiance is consistent among instruments, with maxima and minima occurring at approximately the same wavelengths, the effect of the different bandwidths is also apparent. As the bandwidths of the instruments increases, from USDA to AES to NSF to SERC, the measured spectral irradiance becomes smoother. The maxima and minima measured by the NSF instrument are not as pronounced as they are with the USDA instrument, and virtually no structure is evident with the SERC instrument. The effect of the wider bandwidth of the SERC instrument, combined with the rapid decrease in solar irradiance, is also apparent in Fig. 6.1(b). The irradiance measured by the SERC instrument is greater than that measured by the other instruments at wavelengths shorter than 305 nm because the signal from each filter channel is predominately weighted by the irradiance at wavelengths greater than the center wavelength of that filter. One method for taking this effect into account is to use an effective center wavelength for each filter [9]. However, that approach requires an estimate of the actual solar irradiance, so it will not be discussed further in this paper.

The problem remains of how to compare the solar irradiances measured by instruments with different bandwidths. While deconvolution and spectral synthesis techniques are being investigated, the approach taken for this paper is to convolve the irradiances with a common slit-scattering function. This assumes the instruments are accurately measuring the solar irradiance, so that the convolution is the solar irradiance that would be obtained by a hypothetical instrument with a given slit-scattering function.

The results are presented in order of increasing complexity of the slit-scattering function used in the convolution. In the simplest case, the solar irradiances from the scanning spectroradiometers (AES, EPA, NSF, and USDA) are compared by convolving each irradiance measured by the instrument with a 1 nm FWHM ideal triangular slit-scattering function. The resulting irradiances have spectral structures that are nearly identical to that of the NSF instrument shown in Fig. 6.1. Therefore, the effect of this convolution is that all the instruments have the same 1 nm triangular bandwidth.

**Fig. 6.2:**
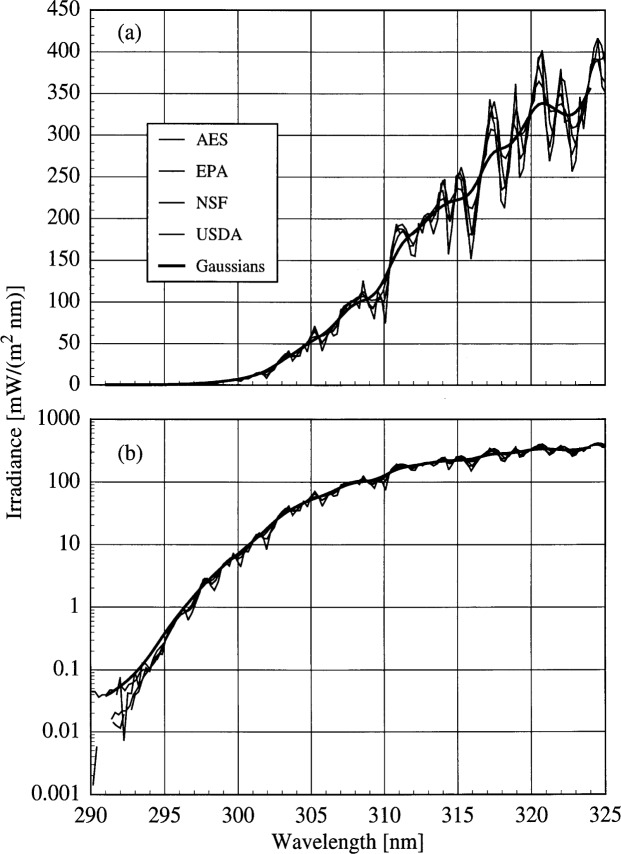
Solar irradiance on a linear scale (a) and on a logarithmic scale (b) as a function of wavelength determined by the instruments indicated in the legend, as well as the average of the irradiances obtained by convolving with Gaussian slit-scattering functions, on day 172 at 17.0 h.

The next level of complexity is to convolve the irradiance measured by one scanning instrument with the slit-scattering functions of all the other scanning instruments, which is equivalent to convolving the true solar spectral irradiance with the slit-scattering functions of all the instruments. This is approximated by convolving the measured irradiance from one instrument with a Gaussian whose bandwidth is the root-sum-square of the nominal bandwidths of the other instruments [10]. For example, the bandwidth of the Gaussian used to convolve the irradiances measured by the AES instrument is 1.2 nm, the root-sum-square of 0.6 nm, 1.0 nm, and 0.3 nm, the nominal bandwidths of the EPA, NSF, and USDA instruments, respectively. The minimum of the Gaussian was set to 10^−5^ to account for the typical measured stray-light rejections of the instruments. This convolution appreciably smoothes the structure of the measured irradiances, as shown in Fig. 6.2, where the irradiances measured by the different instruments are plotted as a function of wavelength, as well as the average of the irradiances convolved with the Gaussian slit-scattering functions.

The final convolution technique allows comparisons among all the instruments. The irradiances measured by the scanning instruments are convolved with the filter transmittances of the SERC instrument. This approach does not require any additional knowledge about the atmosphere, solar spectral irradiance, or radiative transfer. The irradiance *E_j_* at filter channel *j* for each scanning instrument is given by

$$E_{j}=\sum_{i}E(\lambda_{i})\tau_{j}(\lambda_{i})/\sum_{i}\tau_{j}(\lambda_{i})$$ (6.2)

where *i* indexes the wavelength *λ* and *τ_j_* is the filter transmittance for channel *j*. To account for the stray-light rejection of these filters, shown in Fig. 5.2, the minimum filter transmittance was set at 10^−5^. The resulting spectral irradiances are nearly identical to that for the SERC instrument shown in Fig. 6.1.

**Fig. 6.3:**
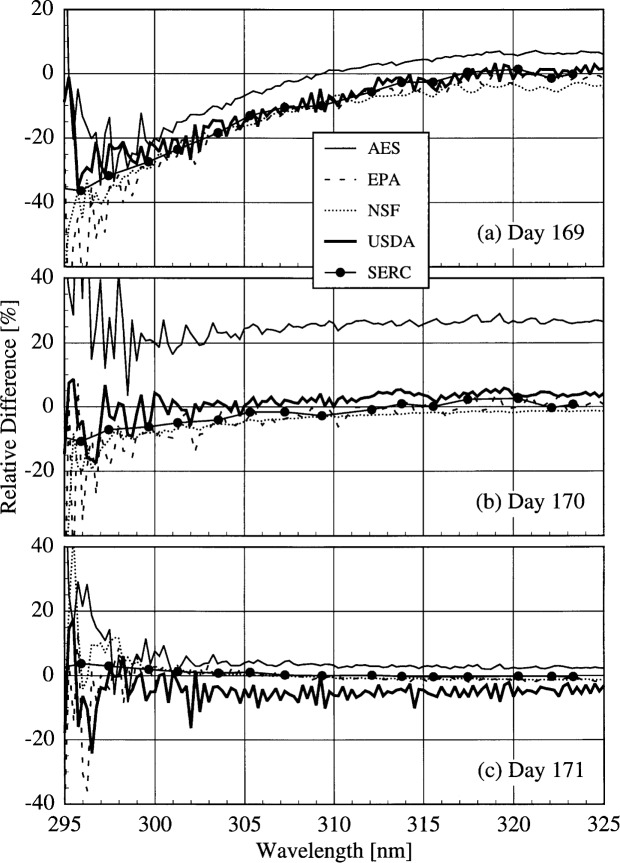
Relative difference between solar irradiances determined on the day indicated in the panel to those determined on day 172, both at 15.0 h, as a function of wavelength for the instruments indicated in the legend.

Since the goal of all the monitoring networks is to detect changes in solar ultraviolet irradiance due to ozone depletion, it is instructive to compare the irradiances measured by each instrument on different days. Since all the instruments were working properly on day 172, and all the instruments were measuring at 15.0 h on each day, the relative differences between the solar irradiances measured on other days to those measured on day 172 were calculated. The results are shown in Fig. 6.3, where the relative difference is plotted as a function of wavelength.

The problem with the PMT voltage of the AES instrument is apparent in Figs. 6.3(a) and 6.3(b), where the relative differences for this instrument are markedly different than those of the other instruments. The drift in the responsivity of the USDA instrument is also noticeable, as the relative differences for this instrument are greater than for the other instruments on day 170, and less than for the others on day 171. The spectral structure of the relative differences for this instrument are indicative of changes in the wavelength calibration. The other three instruments were operating well on all four days, as indicated by the agreement of the relative differences between the instruments and by the lack of any spectral structure.

The results can be understood from the atmospheric conditions at the times of the measurements. Since the total column ozone, from Fig. 4.2, was greater on day 169 than on day 172 at 15.0 h, the relative difference in Fig. 6.3(a) was less than zero for wavelengths shorter than 315 nm. In contrast, both the total column ozone and the turbidity, from Fig. 4.1, remained nearly constant on days 170 to 172 at 15.0 h, resulting in a relative difference close to zero at all wavelengths in Figs. 6.3(b) and 6.3(c).

The value used to quantify the agreement between instruments is the relative standard deviation, given by the standard deviation of the solar irradiances divided by the average irradiance at each wavelength. Since all the instruments performed synchronized spectral scans under nominally identical conditions, they are all assumed to have been exposed to the same spectral irradiance. However, each instrument measured an independent value for the irradiance, and therefore the relative standard deviation of these independent values is used to indicate the agreement between instruments.

**Fig. 6.4:**
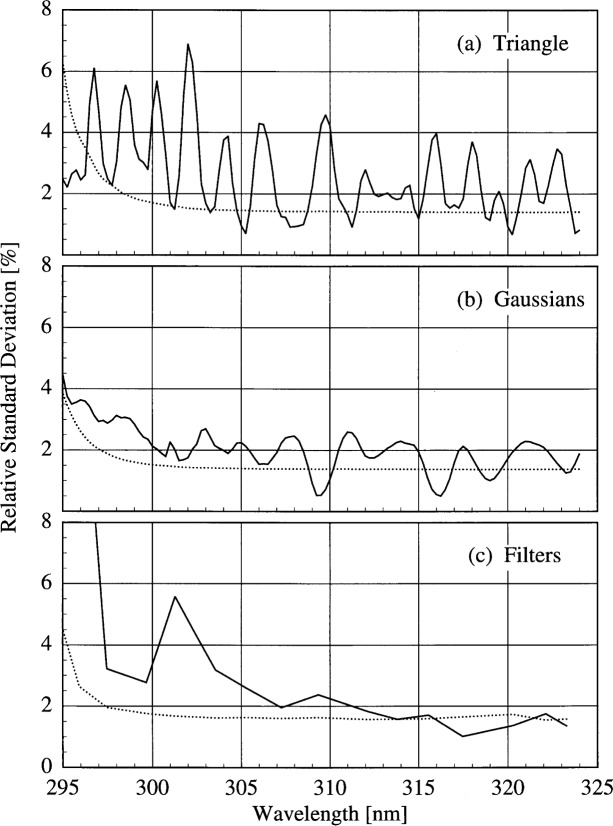
Relative standard deviation as a function of wavelength of the solar irradiances measured by all but the SERC instrument (a) and (b) and by all the instruments (c) on day 172 at 17.0 h convolved with the slit-scattering functions indicated in the panels (solid lines). The dashed lines are the relative standard deviations calculated from the uncertainties of the measurements.

The results presented here focus on the irradiances measured on day 172 at 17.0 h since all the instruments were operating correctly and the sky was clear. These results are representative of those obtained on different days and with different combinations of instruments. The relative standard deviations obtained from the three convolution techniques are the solid lines shown in Fig. 6.4 as a function of wavelength. The dashed lines are the relative standard deviations calculated from the propagated uncertainties in the responsivities and the signals and including a 1 % uncertainty in responsivity due to drift. There are no significant differences between the relative standard deviations shown in Fig. 6.4 and those obtained on different days and with different instrument combinations but using the same convolution techniques, even when the participants were using their own scanning routines on day 171.

The relative standard deviations using the triangular convolution are in the range from 1 % to 6 %, are usually greater than expected from the propagation of uncertainties, and are correlated with the solar spectrum. At those wavelengths where the solar spectrum is changing most rapidly, such as near 310 nm and between 315 nm and 320 nm, the relative standard deviation is large and peaked. This correlation with the solar spectrum suggests that wavelength uncertainties among the instruments are responsible for much of the spectral structure of the relative standard deviation. Indeed, using the same analysis as in Sec. 6.4.2 of Ref. [2] and assuming a 2 % relative standard deviation from all other sources, a wavelength uncertainty of approximately 0.15 nm accounts for the relative standard deviations in Fig. 6.4(a).

In contrast, convolving with Gaussian slit-scattering functions results in relative standard uncertainties that are in the range from 1 % to 3 %, which are approximately the same as those expected from the propagation of uncertainties, and are not correlated with the solar spectrum. The decreased relative standard deviations relative to those obtained with a triangular convolution are primarily a result of convolving with a function which includes all the wavelengths from the spectral scan, and not a limited number as with the triangular convolution. Therefore, convolving with Gaussians is a reasonable method for comparing irradiances measured by instruments with different bandwidths since all the convolved irradiances include the bandwidths of all the instruments.

Finally, the relative standard deviations resulting from convolving with the SERC filter transmittances are in the range from 1 % to 3 %. These values agree well with those expected from the propagation of uncertainties at the longest wavelengths, but increase more rapidly than expected as the wavelength decreases. The relative standard deviations obtained with the Gaussian convolutions are comparable to those obtained by convolving with the filter transmittances.

**Fig. 6.5:**
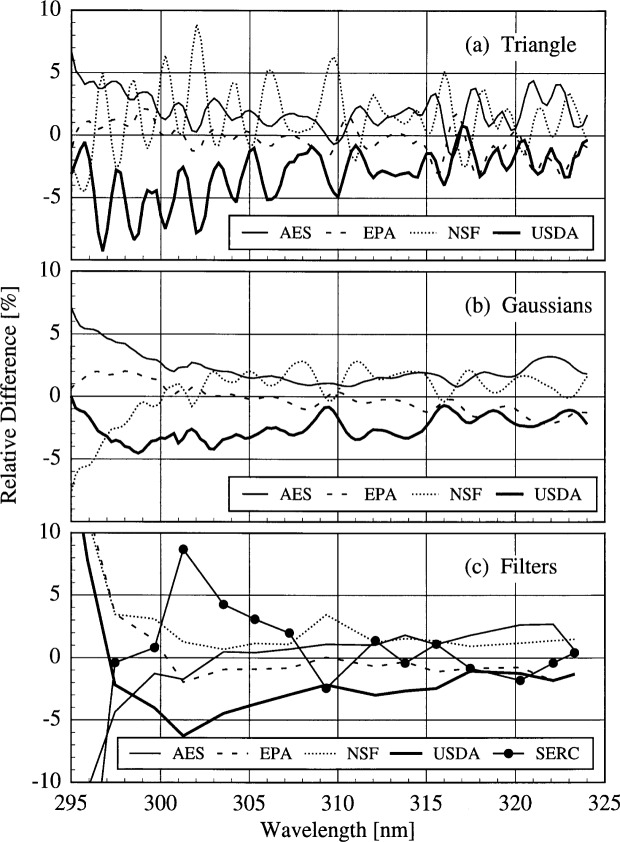
Relative difference between the solar irradiances measured by each instrument to the average irradiance as a function of wavelength on day 172 at 17.0 h. The slit-scattering functions used to convolve the irradiances are indicated in each panel, and the instruments are indicated in the legends.

The relative difference between the convolved irradiance of an individual instrument and the average of all the convolved irradiances on day 172 at 17.0 h is shown in Fig. 6.5 as a function of wavelength. The ratios are generally within ± 5 % for all three convolution techniques. As with the relative standard deviations, the relative differences have more spectral structure with the triangular convolution than with the Gaussian or filter convolutions. On this day, the irradiances measured with the USDA instrument were consistently lower than the average. In contrast, on day 169 they agreed well with the average and on day 170 they were consistently higher. This is a result of the responsivity instability of this instrument detailed in Sec. 5.3.4.

**Fig. 6.6:**
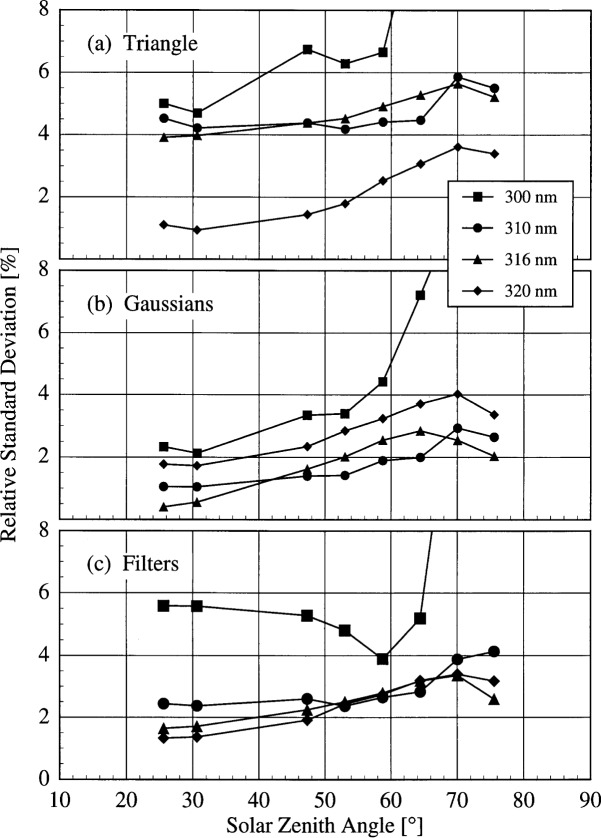
Relative standard deviation as a function of solar zenith angle of the solar irradiances measured at the wavelengths indicated in the legend by all but the SERC instrument (a) and (b) and by all the instruments (c) on day 172 during clear sky conditions convolved with the slit-scattering functions indicated in the panels.

The relative standard deviation of the convolved irradiances at selected wavelengths as a function of solar zenith angle is shown in Fig. 6.6 on day 172 while the sky was clear. The convolution techniques are indicated in the panels. The wavelengths were chosen to be representative of the spectral structure of the relative standard deviation, and for the convolutions with the SERC filter transmittances the wavelengths were chosen to be as close as possible to those given in the legend of Fig. 6.6. As noted above, the relative standard deviations from the convolutions with the Gaussian slit-scattering functions and the filter transmittances are smaller than those from the convolution with the triangular slit-scattering function. The standard deviations generally increase with decreasing solar zenith angle, partly due to the increased uncertainties in the measurements, but also indicating a difference between the instruments, possibly in either the Lambertian quality of the diffusers or the linearity of the detectors.

The synchronized solar scans on the afternoon of day 172 occurred during cloudy conditions. Even so, the relative standard deviations of the irradiances convolved with the triangular and Gaussian slit-scattering functions were similar to those with clear conditions. This indicates that the irradiances measured with the scanning instruments are comparable under a variety of atmospheric conditions. In contrast, the relative standard deviations of the irradiances convolved with the SERC filter transmittances were as large as 20 %. This is a result of the changing conditions during the 7 min of the synchronized scans. While the scanning instruments measured the irradiance at each wavelength only once during the scan, the irradiances from the SERC instrument were averaged over the entire 7 min. Therefore, partly cloudy conditions would result in irradiances measured by the SERC instrument that were markedly different from those measured by the scanning instruments.

The agreement between the solar ultraviolet irradiances measured by the instruments is somewhat dependent on the slit-scattering function used in the convolution. With a triangle, the relative standard deviations of irradiance are in the range from 1 % to 6 %, much of which can be attributed to an uncertainty in the wavelength between the instruments. With functions which are not as sensitive to wavelength uncertainties because the convolutions include the entire wavelength range, the relative standard deviations of irradiance range from 1 % to 3 %. In all cases, the relative standard deviation increases with increasing solar zenith angle. Since the results shown in Figs. 6.4, 6.5, and 6.6 are similar to those obtained on other days and with different instrument combinations, the relative standard deviations of the irradiances measured at this Intercomparison are indicative of the present level of agreement that can be achieved with current monitoring spectroradiometers.

## 7. Conclusions

The 1995 Intercomparison improved upon the techniques used and results obtained at the 1994 Intercomparison, particularly in terms of outdoor instrument characterizations. Spectral scans of the emission lines from Hg and Cd lamps and a HeCd laser were performed both indoors and outdoors. The consistency in the instrument parameters obtained among sources and locations indicate that the intrinsic values of these parameters were measured at this Intercomparison.

The stray-light rejections of the instruments were consistent with those expected for single- and double-grating monochromators and for interference filters. The only exception was the NSF instrument, whose measured stray-light rejection was limited by the dynamic range of the signal. The bandwidths of all the scanning instruments decreased with increasing wavelength, which was attributed to quartz lenses in all the optical paths. The wavelength uncertainties showed some dependence on wavelength.

Results obtained with the internal and external Hg lamps of the NSF instrument were very instructive. The different magnitudes and wavelength dependencies of the bandwidth indicated the necessity of using emission lamps on the same optical path as that used to measure irradiance in order to properly characterize the instrument. In addition, the technique used to determine the wavelength calibration of an instrument from emission lines, either the centroid or the peak signal, can result in different wavelength calibrations and uncertainties. Therefore, a common technique should be adopted by all networks.

Standard lamps calibrated in the horizontal position with the NIST spectral irradiance scale were used to determine the responsivity of each instrument. The technique to align and operate the lamps was greatly improved over that at the 1994 Intercomparison by using a field calibration unit. This unit allowed the lamps to be easily and reproducibly aligned with the diffuser of each instrument and to operate at any time of the day.

The differences between the irradiance scales of the participants and the NIST scale were between − 5 % and + 10 %, which is within the same range as at the 1994 Intercomparison. These results indicate the advantage of using a common irradiance standard for determining instrument responsivities, and that additional efforts need to be made to improve the agreement among the participants’ spectral irradiance scales. The responsivities of the instruments changed upon moving them outdoors, particularly the USDA instrument, and generally remained constant to within ± 5 % outdoors. As with the 1994 Intercomparison, this points to the necessity of performing these determinations at the locations where the instruments are measuring solar irradiance.

Synchronized solar irradiance scans from 290 nm to 325 nm were performed every half-hour for 4 days of the Intercomparison. Because the instruments had different bandwidths, the measured irradiances were convolved to a common bandwidth. The three slit-scattering functions used for the convolutions were chosen for simplicity (triangle), to approximate convolving with the slit-scattering functions of the other instruments (Gaussian), and to include the irradiances measured by the SERC instrument (filter transmittances). The agreement among the convolved irradiances was described by their relative standard deviations.

The scanning procedure of the instruments was not a significant component of the relative standard deviations since values obtained on the day when each instrument used a typical scan routine for its network were similar to those obtained on days when all the scans were synchronized at each wavelength. This conclusion is based upon a clear day, during which the solar irradiance changed slowly with time. It is not expected to remain valid if the irradiance is changing rapidly with time, as during partly cloudy conditions.

The maximum relative standard deviations decreased from 6 % to 3 % and were not correlated with the structure of the solar spectrum as the convolution function included more signals from each scan. Thus, wavelength uncertainties among the instruments of approximately 0.15 nm are a significant component of the relative standard deviations of irradiances obtained by convolving with a triangular slit-scattering function. As demonstrated by the results from the instrument characterizations, the techniques used to calibrate the wavelength of the instruments vary between networks. Therefore, a common technique among the networks for such calibrations should improve the agreement among measured irradiances. In addition, deconvolution techniques should be investigated which will compare the measured to the actual solar spectrum and thereby improve the instrument wavelength calibrations.

The instruments must also be stable. While the responsivities of most instruments change upon movement, they should change as little as possible while the instrument is monitoring solar ultraviolet irradiance. From the 1994 and 1995 Intercomparisons, the responsivities of all the instruments change over several days, usually less than 3 % but for some instruments as much as 10 %. Therefore, efforts need to be made to improve the thermal, mechanical, electrical, and optical stabilities of the instruments. Efforts to improve the Lambertian quality and linearity of the instruments might also decrease the dependence of the relative standard deviations on solar zenith angle.

Overall, the Intercomparison was very successful. The weather cooperated, all the instrument characterizations were able to be performed outdoors, and the instruments operated correctly. The results from the data yielded valuable information about the performance of the instruments, the present level of agreement that can be expected between measured irradiances, and possible improvements in techniques and instruments.

## Figures and Tables

**Table 1.1:** Instruments present during the 1995 North American Interagency Intercomparison of Ultraviolet Monitoring Spectroradiometers

Network	Participating spectroradiometers Instrument	Serial no.
AES	Sci-Tec Brewer MKII	039
EPA	Sci-Tec Brewer MKIV	114
NSF	BSI SUV-100	B-007
SERC	SERC SR-18	UD
USDA	ASRC/RSI UV Spectroradiometer	901

Instrument	Ancillary instruments	Serial no.

Eppley Precision Solar Pyranmometer (Downwelling)		29613
Eppley Precision Solar Pyranmometer (Shaded)		29614
Eppley Precision Solar Pyranmometer (Upwelling)		29616
Eppley Precision Infrared Pyrgeometer		29143
Eppley Precision Infrared Pyrgeometer		29144
Eppley Precision Infrared Pyrgeometer		29149
HF-Cavity Radiometer		
Yankee UVB-1 Radiometer		940401
Yankee UVB-1 Radiometer		940402
Yankee UVB-1 Radiometer		940404
Solar Light UV-Biometer		1886
Solar Light UV-Biometer		1898
Yankee Multi-Filter Rotating Shadowband Radiometer		
Biospherical GUV-511B		9263

Measurement	Meterological instruments	Instrument

Temperature and relative humidity		Vaisala HMP 35C
Wind speed and direction		R. M. Young 05305
Barometric pressure		Vaisala PTB101B

**Table 3.1:** Spectroradiometer specifications

Participant	AES	EPA	NSF	SERC	USDA
Spectroradiometer					
Model	Brewer	Brewer	BSI	SERC	ASRC/RSI
	MKII	MKIV	SUV-100	SR-18	UV Spect.
Serial no.	039	114	B-007	UD	901
F – number	6	6	3.5		5.5
Diffraction grating					
Number	1	1	2		2
Type	plane holographic	plane holographic	concave holographic		plane holographic
Lines per millimeter	1800	1200	1200		3600
Blaze (nm)		250			250
Diffraction order	second	third	first		first
Dispersion	1 nn/mm	1 nm/mm	4 nm/mm		200 nm/rad
PMT	9789QA	9789QA	R-269	R-1657	R-2371HA
Bandwidth(nm)	0.6	0.6	0.95	2 (nominal)	0.3
Step (nm)					
usual	0.5	0.5	0.2, 0.5, 1	2 (nominal)	0.15
finest	0.1	0.1	0.1		0.005
Range (nm)	290 to 325	286 to 363	280 to 620	290 to 324	280 to 400
Diffuser material	Teflon	Teflon	Teflon	Teflon	Spectralon
Weatherproof ?	Yes	Yes	Yes	Yes	No
Automatic ?	Yes	Yes	Yes	Yes	Yes
Temperature					
Stabilized Optics?	No	No	Yes	No	Yes
Stabilized detector ?	No	No	Yes	Yes	Yes
Dark current removed ?	Yes	Yes	Yes	Yes	Yes
Stray light removed ?	Yes	Yes	Yes	No	No
Wavelength registration (nm)	302.3	302.3	296.7		296.7 334.1
Primary lamp (W)	1000	1000	200	1000	
Secondary lamp (W)	50	50	45		

**Table 3.2:** Channel indicator, nominal and actual center wavelength, bandwidth, and maximum transmittance for each filter of SERC instrument UD. Note the order reversal in the designations of the first nine filters. An (^*^) with the maximum transmittance indicates the channel has an additional 0.5 OD neutral-density filter

Channel	Nominal center wavelength (nm)	Actual center wavelength (nm)	Bandwidth (nm)	Maximum transmittance
I	290	289.72	2.25	0.075
H	292	291.59	2.47	0.068
G	294	293.59	2.38	0.090
F	296	295.92	2.30	0.061
E	298	297.45	2.43	0.068
D	300	299.66	2.39	0.060
C	302	301.28	2.24	0.085
B	304	303.54	2.65	0.107^*^
A	306	305.31	2.10	0.104^*^
J	Dark			
K	308	307.26	2.55	0.091^*^
L	310	309.34	2.21	0.121^*^
M	312	312.13	2.18	0.096^*^
N	314	313.80	2.60	0.074^*^
O	316	315.56	2.60	0.076^*^
P	318	317.47	2.44	0.112^*^
Q	320	320.28	2.77	0.110^*^
R	322	322.12	2.46	0.114
S	324	323.30	2.39	0.096
T	Dark			

**Table 5.1:** Dates, lamps, times, and instrument temperatures of spectral scans determining responsivity

Instrument	Day	Lamp	Time (h)	Instrument temperature (°C)
AES	165	S-702	20.34	38.2
		S-789	21.00	37.9
	166	OS-27	0.08	36.9
		F-332	0.91	38.3
		S-789	22.59	32.7
	167	U-5	15.78	28.4
		S-702	16.38	31.2
		OS-27	18.45	34.1
		F-332	19.61	37.6
		S-702	20.82	36.8
	168	U-5	16.30	29.6
		S-702	17.18	32.9
		S-789	17.81	34.4
	170	S-789	1.00	40.9
		S-702	1.46	39.4
		U-5	1.93	37.9
		F-332	2.53	33.3
	171	F-332	0.08	45.4
		U-5	1.13	43.1
	172	U-5	2.15	38.7
		F-332	21.80	40.6
		S-789	22.80	41.3
		U-5	23.41	42.0
		5-702	23.89	42.4
EPA	166	327	18.36	25.4
		05-27	21.58	30.8
		F-332	22.57	31.4
	169	327	21.63	35.1
		326	22.33	36.2
		328	22.85	36.8
		329	23.43	36.4
	170	F-332	0.01	35.9
		327	23.91	38.5
	171	326	0.52	37.8
		330	1.02	37.2
		328	1.56	36.4
		5-789	21.64	33.6
		U-5	22.33	32.9
	172	329	3.34	26.8
		329	22.55	33.6
		F-332	23.47	36.4
	173	329	20.38	28.1
		330	20.98	28.4
NSF	167	OS-27	23.73	
	168	F-332	0.60	
		45W	1.68	
		M-761	2.55	
		M-767	3.38	
	170	F-332	1.25	
		M-761	2.68	
		M-767	3.35	
		45W	3.95	
	173	F-332	16.12	
		M-767	17.43	
		M-761	19.08	
		45W	19.67	
SERC	165	OS-27	19.07	34.4
		F-332	20.05	32.1
	166	OS-27	16.97	32.9
		F-332	18.02	33.2
	169	F-332	21.85	36.2
	170	OS-27	22.18	35.2
		F-332	22.73	32.8
	173	F-332	17.10	39.6
		OS-27	20.45	37.7
		F-332	20.97	34.5
USDA	167	OS-27	11.25	
		F-332	12.56	
	169	F-332	22.67	
	172	F-332	22.67	

**Table 5.2:** Relative standard uncertainties from all components during responsivity measurements at selected wavelengths

Component	Wavelength (nm)	AES	Relative standard uncertainty (%)			
			EPA	NSF	SERC	USDA
Lamp						
Irradiance	290	1.06	1.06	1.06	1.06	1.06
	320	0.85	0.85	0.85	0.85	0.85
	350	0.80	0.80	0.80	0.80	0.80
Size		0.09	0.09	0.03	0.02	0.05
Goniometry		0.46	0.46	0.30	0.27	0.36
Current	290	0.02	0.02	0.02	0.02	0.02
(random)	320	0.02	0.02	0.02	0.02	0.02
	350	0.02	0.02	0.02	0.02	0.02
Current	290	0.07	0.07	0.07	0.07	0.07
(systematic)	320	0.06	0.06	0.06	0.06	0.06
	350	0.06	0.06	0.06	0.06	0.06
Alignment						
Perpendicular		0.29	0.29	0.29	0.29	0.29
Center		0.09	0.09	0.09	0.09	0.09
Distance		0.23	0.23	0.23	0.23	0.23
Instrument						
Wavelength	290	0.31	0.02	0.46	0.19	0.13
	320	0.16	0.03	0.33	0.16	0.04
	350		0.03	0.22		0.02
Signal	290	0.47	0.27	0.60	0.11	0.68
	320	0.22	0.18	0.17	0.64	0.30
	350		0.24	0.16		0.15
Combined						
Random	290	0.47	0.27	0.60	0.11	0.68
	320	0.22	0.18	0.17	0.64	0.30
	350		0.24	0.16		0.15
Systematic	290	1.26	1.22	1.30	1.18	1.19
	320	1.06	1.04	1.07	0.98	1.00
	350		1.00	0.98		0.96

**Table 5.3:** Lamps, times, and temperature changes for responsivitiy relative differences used in the figures

Figure	Lamp	Numerator Day	Time (h)	Lamp	Denominator Day	Time (h)	Temperature change (°C)
5.8(a)	F-332	166	0.91	S-702	165	20.34	+ 0.1
5.8(a)	F-332	166	0.91	S-789	165	21.00	+ 0.4
5.8(b)	F-332	166	22.57	327	166	18.36	+ 6.0
5.8(c)	F-332	168	0.60	M-761	168	2.55	
5.8(c)	F-332	168	0.60	M-767	168	3.38	
5.9(a)	F-332	170	2.53	U-5	170	1.93	− 4.6
5.9(a)	F-332	172	21.80	S-789	172	22.80	− 0.7
5.9(a)	F-332	172	21.80	U-5	172	23.41	− 1.4
5.9(a)	F-332	172	21.80	S-702	172	23.89	− 1.8
5.9(b)	F-332	170	0.01	327	169	21.63	+ 0.8
5.9(b)	F-332	170	0.01	326	169	22.33	− 0.3
5.9(b)	F-332	170	0.01	328	169	22.85	− 0.9
5.9(c)	F-332	170	1.25	M-761	170	2.68	
5.9(c)	F-332	170	1.25	M-767	170	3.35	
5.9(c)	F-332	173	16.12	M-761	173	19.08	
5.10(a)	F-332	170	2.53	F-332	166	0.91	− 5.0
5.10(b)	F-332	170	0.01	F-332	166	22.57	+ 4.5
5.10(c)	F-332	170	1.25	F-332	168	0.60	
5.10(d)	F-332	169	21.85	F-332	165	20.05	+ 4.1
5.10(d)	F-332	173	17.10	F-332	170	22.73	+ 6.8
5.10(e)	F-332	169	22.67	F-332	167	12.56	
5.11(a)	S-702	170	1.46	S-702	167	16.38	+ 2.6
5.11(a)	S-789	170	1.00	S-789	166	22.59	+ 8.2
5.11(a)	U-5	170	1.93	U-5	167	15.78	+ 9.5
5.11(b)	327	169	21.63	327	166	18.36	+ 9.7
5.11(c)	M-761	170	2.68	M-761	168	2.55	
5.11(c)	M-767	170	3.35	M-767	168	3.38	
5.11(c)	45W	170	3.95	45W	168	1.68	
5.12(a)	F-332	172	21.80	F-332	170	2.53	+ 7.3
5.12(b)	F-332	172	23.47	F-332	170	0.01	+ 0.5
5.12(c)	F-332	173	16.12	F-332	170	1.25	
5.12(d)	F-332	173	17.10	F-332	169	21.85	+ 3.4
5.12(e)	F-332	172	22.67	F-332	169	22.67	
5.13(a)	U-5	171	1.13	U-5	170	1.93	+ 5.2
5.13(a)	U-5	172	2.15	U-5	171	1.13	− 4.4
5.13(a)	U-5	172	23.41	U-5	172	2.15	+ 3.3
5.13(b)	326	171	0.52	326	169	22.33	+ 1.6
5.13(b)	327	170	23.91	327	169	21.63	+ 3.4
5.13(b)	328	171	1.56	328	169	22.85	− 0.4
5.13(c)	M-761	173	19.08	M-761	170	2.68	
5.13(c)	45W	173	19.67	45W	170	3.35	

**Table 6.1:** Days and times, indicated by an “X,” at which participating instruments were performing synchronized spectral scans of solar ultraviolet irradiance

Day	Time (h]	AES	Participating instruments			USDA
			EPA	NSF	SERC	
169	13.0		X	X		X
	13.5		X	X		X
	14.0		X	X	X	X
	14.5		X	X	X	X
	15.0		X	X	X	X
	15.5		X	X	X	X
	16.0		X	X	X	X
	16.5		X	X	X	X
	17.0		X	X	X	X
	17.5		X	X	X	X
	18.0		X	X	X	X
	18.5		X	X	X	X
	19.0		X	X	X	X
	19.5		X	X	X	X
	20.0		X	X	X	X
	20.5		X	X	X	X
	21.0		X	X		X
	21.5					X
	22.0					X
170	13.0		X	X	X	
	13.5		X	X	X	
	14.0		X	X	X	
	14.5		X	X	X	X
	15.0		X	X	X	X
	15.5		X	X	X	X
	16.0		X	X	X	X
	16.5		X	X	X	X
	17.0		X	X	X	X
	17.5		X	X	X	X
	18.0		X	X	X	X
	18.5		X	X	X	X
	19.0		X	X	X	X
	19.5		X	X	X	X
	20.0		X	X	X	X
	20.5		X	X	X	X
	21.0		X	X	X	X
	21.5					X
	22.0					X
171	13.0		X	X		X
	13.5		X	X		X
	14.0		X	X	X	X
	14.5		X	X	X	X
	15.0		X	X	X	X
	15.5		X		X	X
	16.0		X	X	X	X
	16.5			X	X	
	17.0	X	X	X	X	
	17.5	X	X	X	X	
	18.0	X	X	X	X	
	18.5	X	X	X	X	
	19.0	X	X	X	X	
	19.5	X	X	X	X	
	20.0	X	X	X	X	
	20.5	X	X	X	X	
	21.0	X	X		X	
	21.5				X	
	22.0				X	
172	13.0	X	X	X	X	X
	13.5	X	X	X	X	X
	14.0	X	X	X	X	X
	14.5	X	X	X	X	X
	15.0	X	X	X	X	X
	15.5	X	X	X	X	X
	16.0		X	X	X	X
	16.5	X	X	X	X	X
	17.0	X	X	X	X	X
	17.5	X	X	X	X	X
	18.0	X	X	X	X	X
	18.5	X	X	X	X	X
	19.0	X	X	X	X	X
	19.5	X	X	X	X	X
	20.0	X	X	X	X	X
	20.5	X	X	X		X
	21.0	X	X	X		X

**Table 6.2:** Days and times of responsivity scans used to calculate solar irradiances

Day of solar scan	AES	Day/Time of responsivity scan		USDA
		EPA	SERC	
169		170/0.01	169/21.85	169/22.67
170		170/0.01	169/21.85	169/22.67
171	172/21.80	172/23.47	173/17.10	172/22.67
172	172/21.80	172/23.47	173/17.10	172/22.67

## Footnotes

1 Certain commercial equipment, instruments, or materials are identified in this paper to foster understanding. Such identification does not imply recommendation or endorsement by the National Institute of Standards and Technology, nor does it imply that the materials or equipment identified are necessarily the best available for the purpose.

## 8. Appendix A. Attendees

The following people attended the 1995 North American Interagency Intercomparison of Ultraviolet Monitoring Spectroradiometers, and are grouped by function and network.

**Table:**

COORDINATORS		
National Institute of Standards and Technology (NIST)		
Ambler Thompson	(301) 975-2333	ambler@enh.nist.gov
Edward Early	(301) 975-2343	early@enh.nist.gov
Carol Johnson	(301) 975-2322	cjohnson@nist.gov
Fax	(301) 840-8551	
NIST		
Bldg. 220, Rm. A-320		
Gaithersburg, MD 20899-0001		
National Oceanic and Atmospheric Administration (NOAA)		
John DeLuisi	(303) 497-6083	deluisi@srrb.noaa.gov
Patrick Disterhoft	(303) 497-6355	dister@srrb.noaa.gov
Fax	(303) 497-6546	
NOAA R/E/ARx1		
325 Broadway		
Boulder, CO 80303		
PARTICIPANTS		
Atmospheric Environment Service (AES)		
David Wardle	(416) 739-4632	dwardle@dow.on.doe.ca
Edmund Wu	(416) 739-4256	ewu@dow.on.dow.ca
Fax	(416) 739-4281	
Environment Canada		
4905 Dufferin Street		
Toronto, ON M3H 5T4 Canada		
Environmental Protection Agency (EPA)		
Yongchen Sun	(706) 542-1396	ysun@uga.cc.uga.edu
Wanfeng Mou	(706) 542-6768	wmou@hal.physast.uga.edu
Fax	(706) 542-2492	
University of Georgia		
Dept. of Physics and Astronomy		
University of Georgia		
Athens, GA 30602		
National Science Foundation (NSF)		
Timothy Lucas	(619) 686-1888	lucas@biospherical.com
Tanya Mestechkina	(619) 686-1888	tanya@biospherical.com
Fax	(619) 686-1887	
Biospherical Instruments, Inc.		
5340 Riley Street		
San Diego, CA 92110-2621		
Smithsonian Environmental Research Center (SERC)		
Douglass Hayes	(301) 261-4190 ext. 131	hayes@serc.si.edu
Fax	(301) 261-7954	
Smithsonian Environmental Research Center of the Smithsonian Institution		
P.O. Box 28		
Edgewater, MD 21037		
United States Department of Agriculture (USDA)		
Lee Harrison	(518) 442-3811	lee@solsunl.asrc.albany.edu
Jerry Berndt	(518) 442-3788	jerry@solsunl.asrc.albany.edu
Fax	(518) 442-3867	
Atmospheric Sciences Research Center		
State University of New York at Albany		
100 Fuller Road		
Albany, NY 12205		

## 9. Appendix B. Upper-Air Conditions

The tropospheric wave pattern over North America during the 3 days before the Intercomparison was dominated by an “omega block,” where a sharp ridge is flanked to the east and west by deep troughs. Over North America, the narrow central ridge extended from Texas to southern Canada. The eastern component of this pattern was an extension of a deep trough that had been entrenched over northern Quebec for several months. On the morning of day 167, the center of the cut-off low in the western United States had reached as far south as southern California, and by the next morning it had drifted east in extreme southern Nevada and had weakened. At that time the low was stacked vertically above 85 kPa through the depth of the troposphere with nearly concentric height and vorticity isopleths at 50 kPa. These characteristics indicated that it was a slow-moving and nondeveloping or decaying system.

As the closed low moved into Nevada, the axis of the blocking ridge drifted eastward into Iowa. With this, the mid- to upper-tropospheric flow over the Front Range backed from southwesterly to southerly, which was conducive to strong thunderstorm activity. By late afternoon on day 168, the low over Nevada had weakened to the point where it opened and was absorbed by a stronger trough moving on to the northwest coast. As the decaying trough was being assimilated, it assumed the form of a shortwave in the southwesterly flow of the approaching trough. During the afternoon of day 168, the axis of maximum vorticity advection associated with this shortwave was over New Mexico. Strong thunderstorms were triggered that evening over the Front Range as this perturbation propagated north over Colorado.

By the morning of day 169, both middle-level troughs of the omega block had propagated north. In the east, the southern periphery of the trough over eastern Canada withdrew as far north as New England, but also left a weak “orphan” closed low behind in the southeast United States. In the west, the center of the approaching trough was still off the northwest coast, and the maximum vorticity of the remnant short wave, that triggered thunderstorms over the Front Range the night before, was over Wyoming. Thus, on the afternoon of day 169, the northern Front Range was in descending motion on the backside of the short wave. The Denver morning sounding showed significant cooling and drying between the surface and 50 kPa on the backside of this wave. This, combined with the maintenance of a strong inversion around 50 kPa by subsidence, greatly diminished the potential for thunderstorms over the Front Range. The only storms to occur in the region were on the eastern plains of Colorado along a long weak north-south oriented cold front that emanated from a surface low in Saskatchewan. That boundary was instrumental in keeping the high surface moisture on the eastern plains, well to the east of the Front Range.

Over the next 24 hours the center of the approaching trough moved onshore into Oregon. Its slow eastward progress acted in concert with the propagation of the short wave into Canada to allow high pressure to envelop eastern Colorado. Also, like the day before, there was a large surface moisture gradient between the Front Range and eastern plains. The circulation around a small surface low analyzed in the late afternoon over Denver directed high surface moisture from the east to the northeastern plains of Colorado, the Nebraska panhandle, and southern Wyoming. In the Denver area, continued dry conditions in the lower half of the troposphere, and the strengthening of the inversion between 40 kPa and 50 kPa reduced the potential for thunderstorms. The middle-level inversion was fortified through a combination of cooling by dry adiabatic lifting at the top of the daytime boundary (the base of the inversion) and warming above 50 kPa by subsidence. The few isolated thunderstorms that occurred that evening were over northeastern Colorado and over the Nebraska panhandle, where the greatest low-level moisture advection was focused.

The weather near Boulder on day 171 was similar to that of the previous day. The trough over the western United States was weakening and became pinched between the sharp ridge to the east and another approaching closed low south of Alaska. This pincer action forced the western United States trough to elongate along its north-south axis. Concurrently, the large surface high that had dominated the region east of the Rocky Mountains for the past three days began to weaken as low pressure approached from the west. However, northeastern Colorado remained far from the midtropospheric low, now centered over Idaho. Along the Front Range the surface conditions were hot and dry with high temperatures above 30 °C and dew point temperatures near 4 °C. Also, like the day before, there was a sharp moisture gradient across eastern Colorado. The surface pressure still maintained the small low pressure center over the Denver area, but with no discernible frontal boundaries. Southeasterly flow within the northeast quadrant of this low continued to funnel warm moist air from the High Plains north of Denver into extreme northeastern Colorado and southern Wyoming. The subsidence inversion above 50 kPa decayed during the day, but extreme dryness below 50 kPa stifled convection over Denver. Again, the strongest thunderstorm activity that evening occurred to the north of the Denver area, where the advection of surface moisture was greatest.

From day 169 to 171, the trough over Quebec moved out and was replaced by an intensifying closed low that emerged from the Arctic. This acted to reform the omega- block pattern, although much farther north than before the Intercomparison. On the evening of day 171, the sharp ridge between the two closed lows extended deep into north-central Canada, with its axis skirting the western shore of Hudson Bay. This ridge prevented the weak trough over the western United States from propagating eastward. During the daylight on day 172, a short-wave rotating around the western United States trough passed over Colorado. This appears to have intensified the surface low in eastern Colorado, which had drifted slightly south over the past day, causing surface easterlies and moisture advection to increase across the northeastern part of the state. Still, however, this surface low had no associated boundaries; the nearest surface front on the east side of the Rocky Mountains was well to the north in central South Dakota and northern Wyoming. With the disappearance of the middle-level subsidence inversion and the increase in low-level moisture over the northern Front Range, the potential for afternoon thunderstorms increased. By early afternoon, convective storms broke out along the Front Range and made for the first cloudy afternoon of the Intercomparison.

## 10. Appendix C. Wavelength Calibration and the Use of the Centroid

The measurement equation for irradiance is given by

$$S(\lambda_{0})=\int E(\lambda )R(\lambda ,\lambda_{0})d\lambda ,$$ (C.1)

where *λ* is the wavelength, *λ*_0_ is the wavelength setting of the instrument, *E*(*λ*) is the spectral irradiance of the source, and *R*(*λ*, *λ*_0_) is the responsivity function of the instrument. The responsivity function is assumed to depend only on the wavelength and not on other parameters, such as polarization, time, or magnitude of the irradiance. Also, this function is assumed to be relatively sharply peaked in the vicinity of *λ*_0_. As written, the first variable in *R*(*λ*, *λ*_0_) covers the entire wavelength range over which the responsivity function is finite, while the second variable is fixed by the instrument. At this point, *λ*_0_ is arbitrary. The succeeding analysis will yield the best value for *λ*_0_ subject to certain constraints and assumptions.

The spectral irradiance responsivity of an instrument is always determined by measuring the signal from a source of known spectral irradiance *E*_K_(*λ*). For this calibration, Eq. (C.1) becomes

$$S_{K}(\lambda_{0})=\int E_{K}(\lambda )R(\lambda ,\lambda_{0})d\lambda .$$ (C.2)

Assume that this is equivalent to removing the irradiance from the integral and assigning it a value at the wavelength *λ*_0_, yielding

$$S_{K}(\lambda_{0})=E_{K}(\lambda_{0})\int R(\lambda ,\lambda_{0})d\lambda .$$ (C.3)

The integral of the responsivity function is now the spectral irradiance responsivity of the instrument.

When a source with an unknown spectral irradiance *E*_U_(*λ*) is measured by the instrument, Eq. (C.1) becomes

$$S_{U}(\lambda_{0})=\int E_{U}(\lambda )R(\lambda ,\lambda_{0})d\lambda .$$ (C.4)

Again, assuming that the irradiance can be removed from the integral yields

$$S_{U}(\lambda_{0})=E_{U}(\lambda_{0})\int R(\lambda ,\lambda_{0})d\lambda .$$ (C.5)

Dividing Eq. (C.5) by Eq. (C.3) and rearranging to solve for *E*_U_(*λ*_0_) yields

$$E_{U}(\lambda_{0})=E_{K}(\lambda_{0})\frac{S_{U}(\lambda_{0})}{S_{K}(\lambda_{0})}.$$ (C.6)

Substituting the expressions for *S*_K_(*λ*_0_) and *S*_U_(*λ*_0_) from Eqs. (C.2) and (C.4) and rearranging yields the equation that must be satisfied to justify removing the irradiances from the integrals and assigning their values at *λ*_0_, namely

$$\frac{E_{U}(\lambda_{0})}{E_{K}(\lambda_{0})}=\frac{\int E_{U}(\lambda )R(\lambda ,\lambda_{0})d\lambda }{\int E_{K}(\lambda )R(\lambda ,\lambda_{0})d\lambda }.$$ (C.7)

In general, there is limited knowledge about *E*_U_(*λ*), so to solve Eq. (C.7) the irradiances are approximated by Taylor series expansions about *λ*_0_, resulting in

$$E_{K}(\lambda )=a_{0}+a_{1}(\lambda -\lambda_{0})+a_{2}{\left(\lambda -\lambda_{0}\right)}^{2}+\ldots \text{and}$$ (C.8a)

$$E_{U}\left(\lambda \right)=b_{0}+b_{1}\left(\lambda -\lambda_{0}\right)+b_{2}{\left(\lambda -\lambda_{0}\right)}^{2}+\ldots .$$ (C.8b)

Using only the constant terms in Eqs. (C.8) trivially satisfies Eq. (C.7) for any value of *λ*_0_. Including the linear terms, Eq. (C.7) can be solved for *λ*_0_ with the result

$$\lambda_{0}=\frac{\int \lambda R(\lambda ,\lambda_{0})d\lambda }{\int R(\lambda ,\lambda_{0})d\lambda }.$$ (C.9)

Including the quadratic terms results in an equation for *λ*_0_ that is not soluble without knowing the coefficients of the expansions. Therefore, the best that can be done to satisfy Eq. (C.7), given limited knowledge of *E*_U_(*λ*), is to assign the centroid wavelength *λ*_0_, given by Eq. (C.9), as the wavelength of the instrument. This method is preferable to other calculations of *λ*_0_, for example the peak of *R*(*λ*, *λ*_0_) or the average of the half-maximum wavelengths of *R*(*λ*, *λ*_0_), since it satisfies Eq. (C.7).

For a monochromator, the wavelength setting is always expressed in terms of a mechanical unit, *x*. Commonly, *x* is either motor steps or encoder units. Assuming a linear relation between wavelength and the mechanical unit over the wavelength range for which the responsivity function is appreciable,

$$\lambda =c_{0}+c_{1}x,$$ (C.10)

and substituting into Eq. (C.9) yields

$$x_{0}=\frac{\int xR(x,x_{0})dx}{\int R(x,x_{0})dx}.$$ (C.11)

Therefore, centroids can be calculated in terms of either wavelength or mechanical units.

Line sources, such as emission lamps or lasers, are used to calibrate the wavelength of a monochromator. For the purposes here, the lines are assumed to be at only a single wavelength, *λ*_0_, so that the spectral irradiance is given by a Dirac delta function, namely

$$E(\lambda )=E_{1}\delta (\lambda -\lambda_{1}),$$ (C.12)

where *E*_1_ is the irradiance of the line. Again assuming the linear relation given by Eq. (C.10), Eq. (C.12) becomes

$$E(x)=E_{1}\delta (x-x_{1}).$$ (C.13)

Using the mechanical unit in Eq. (C.1) and substituting Eq. (C.13) for the spectral irradiance yields

$$S(x_{o})=E_{1}R(x_{1},x_{0}).$$ (C.14)

Note that *x*_0_, the wavelength setting of the monochromator in mechanical units, is changing as the line is scanned, so in Eq. (C.14)*R*(*x*_1_, *x*_0_) is the value of the responsivity function at wavelength *x*_1_ at monochromator setting *x*_0_.

The centroid of the signals, *x*_c_, from the scan of an emission line is given by

$$x_{c}=\frac{\int xS(x)dx}{\int S(x)dx},$$ (C.15)

which, upon substituting Eq. (C.14) for the signals reduces to

$$x_{c}=\frac{\int xR(x_{1},x)dx}{\int R(x_{1},x)dx}.$$ (C.16)

Equation (C.16) is the same as Eq. (C.11) above, except that the integration is over the second variable of the responsivity function rather than the first. In Eq. (C.11)*x*_0_ is fixed, while in Eq. (C.16)*x*_1_, is fixed. The two equations are equivalent if *R*(*x*, *x*_0_) is invariant in *x*_0_, so that it depends only on the difference *x* − *x*_0_. Then, *R*(*x*_1_, *x*) = *R*(*x*_1_ − *x*) = *R*[−(*x* − *x*_1_,)] = *R*(*x* − *x*_0_), and therefore *R*(*x*_1_, *x*) is simply a reflection of *R*(*x*, *x*_0_) about *x*_0_.

Extending this analysis to the case where the emission lines are unresolved by the instrument,

$$E(x)=\sum_{k}E_{k}\delta (x-x_{k}),$$ (C.17)

and Eq. (C.14) becomes

$$S(x)=\sum_{k}E_{k}R(x_{k},x),$$ (C.18)

where *k* indexes the unresolved lines. Substituting Eq. (C.18) into Eq. (C.15), the centroid of the mechanical units is

$$x_{c}=\frac{\sum_{k}E_{k}\int xR(x_{k},x)dx}{\sum_{k}E_{k}\int R(x_{k},x)dx}.$$ (C.19)

Assuming again that *R*(*x_k_*, *x*) is invariant with respect to *x_k_*, so that the integrals are all the same, *R*(*x_k_*, *x*) is replaced by *R*(*x*′, *x*) for all *x_k_* and Eq. (C.19) becomes

$$x_{c}=\frac{\int xR(x^{\prime },x)dx}{\int R(x^{\prime },x)dx},$$ (C.20)

which is the same as Eq. (C.16).

The centroid of the lines in terms of wavelength is given by

$$\lambda_{c}=\frac{\sum_{k}\lambda_{k}E_{k}}{\sum_{k}E_{k}},$$ (C.21)

where *λ_k_* and *E_k_* are the wavelength and irradiance of the lines indexed by *k*. Each mechanical centroid, given by Eq. (C.15), corresponds to a wavelength centroid, given by Eq. (C.21). The wavelength centroids are fit as a function of mechanical centroids to yield the wavelength calibration, *λ* = *f*(*x*), of the instrument.

Summarizing, to remove the spectral irradiance *E*(*λ*) from the integral of the measurement equation, Eq. (C.1), and assign it a value at wavelength *λ*_0_, Eq. (C.7) must be satisfied. For the most general case, the linear term in a Taylor series expansion of *E*(*λ*) forces *λ*_0_ to be the centroid of the responsivity function *R*(*λ*, *λ*0), given by Eq. (C.9). Note that using the centroid to specify the wavelength *λ*_0_ of *R*(*λ*, *λ*_0_) is also applicable to instruments that use filters instead of a monochromator to select the wavelengths of light to be detected. The responsivity function in Eq. (C.1) is replaced by the filter transmittance *τ* (*λ*, *λ*_0_), where *λ*_0_ is fixed for each filter, and the analysis proceeds as before, with the centroid of the filter transmittance being the proper choice for *λ*_0_.

The wavelength *λ* of an instrument is a function of a mechanical unit *x* specifying a physical position of the monochromator, so that *λ* = *f*(*x*). The function *f* is ideally linear in *x* over the entire wavelength range, but is commonly not. To obtain *f*(*x*), *R*(*λ*, *λ*_0_) is assumed to be sharply peaked about *λ*_0_. Within this limited region, *f*(*x*) is assumed to be linear, so that the wavelength centroid given by Eq. (C.9) is comparable to the mechanical unit centroid given by Eq. (C.11). The centroid of an emission line, or of multiple unresolved lines, is calculated in terms of mechanical units by Eq. (C.15). For multiple unresolved lines, *R*(*x*, *x*_0_) must be assumed to be invariant with respect to *x*_0_ over the wavelength where the receiving aperture is described by polar coordinates *r* and *θ* and the filament is described by cylindrical coordinates *R*_f_, *y*, and *ϕ*. The filaments of FEL-type lamps have a height *H* = 2.0 cm and a radius *R*_f_ = 0.25 cm, and the distance *D* = 50.3 cm. The expression for *T*(*R*) is solved numerically at different values range of the emission lines. Finally, *R*(*x*, *x*_0_) must depend only on the difference *x* − *x*_0_ over the range of *x* for which it is sharply peaked, so that *R*(*x*_1_, *x*) is simply a reflection of *R*(*x*, *x*_0_) about *x*_0_. Fitting the wavelength centroids *λ*_c_ from Eq. (C.21) as a function of the corresponding mechanical unit centroids *x*_c_ yields the wavelength calibration *λ* = *f*(*x*).

## 11. Appendix D. Responsivity Uncertainty Analysis

### D.1. General

The components of uncertainty are conveniently divided among those from the lamp, the alignment of the lamp with the diffuser, and the instrument. The expressions used to calculate the relative standard uncertainties resulting from these components are presented below.

The spectral irradiance of the standard lamps (OS-27 and F-332) has an uncertainty associated with it. The uncertainties in the primary standard lamps obtained from FASCAL are propagated through, along with the experimental uncertainties in the calibration of the secondary lamps, to final combined standard uncertainties in the irradiance of the standard lamps. These uncertainties arise from both random and systematic effects, and are combined into a single relative standard uncertainty.

The spectral irradiance of the standard lamps is determined for a receiving aperture area of 1 cm^2^. However, the diffusers of the instruments at the Intercomparison have areas larger than this. There is therefore an uncertainty in irradiance from the distribution of flux over the larger area, which arises from a systematic effect with an assumed Gaussian probability distribution. The geometrical arrangement is described by a circle of radius *R* (for the receiving aperture) and a cylinder of radius *R*_f_ and height *H* (for the lamp) centered a distance *D* along the normal from the center of the circle. The throughput *T*(*R*) of this geometry is given by

$$T(R)=\int_{0}^{R}dr\int_{0}^{2\pi }d\theta \int_{-H/2}^{H/2}dy\int_{-\pi /2}^{\pi /2}d\phi \frac{R_{f}r(D-R_{f}\cos\phi )(D\cos\phi -R_{f}+r\sin\phi \sin\theta )}{{\left[{\left(r\sin\theta -R_{f}\sin\phi \right)}^{2}+{\left(r\cos\theta -y\right)}^{2}+{\left(D-R_{f}\cos\phi \right)}^{2}\right]}^{2}}$$ (D.1)

of *R*. The throughput for a 1 cm^2^ receiving aperture is given by *T*(*R*_0_), where *R*_0_ = 0.564 cm. Assuming that this throughput applies to receiving apertures of larger areas, the throughput *T*’(*R*) for a receiving aperture of radius *R* is given by

$$T^{\prime }(R)=T(R_{0}){\left(\frac{R}{R_{0}}\right)}^{2}.$$ (D.2)

The relative standard uncertainty in irradiance *u*_s_(*E*)/*E* due to the size of the receiving aperture is given by

$$\frac{u_{s}(E)}{E}=\frac{T(R)-T^{\prime }(R)}{T(R)}=\frac{T(R)}{T(R_{0})}{\left(\frac{R_{0}}{R}\right)}^{2}-1.$$ (D.3)

Using the values for *T*(*R*) calculated from Eqs. (D.1) and (D.2) in Eq. (D.3) and fitting with a second-order polynomial yields an empirical expression for *u*_s_(*E*)/*E* given by

$$\begin{array}{c} \frac{u_{s}(E)}{E}=1.2665\times 10^{-4}\\ -3.0508\times 10^{-6}/\text{cm}\cdot R-3.9474\times 10^{-4}/{\text{cm}}^{2}\cdot R^{2}. \end{array}$$ (D.4)

The expressions derived in the preceding paragraph assume that the flux from the lamp is independent of direction. However, actual lamps have a goniometric distribution of irradiance due to the non-uniformity of coiled-coil filaments. The relative standard uncertainty due to this distribution arises from a systematic effect with an assumed Gaussian probability distribution. Let *g*_avg_(2°) be the average goniometric distribution of irradiance relative to the irradiance at 0° for pitch and yaw angles less than or equal to 2°. For most standard lamps, *g*_avg_ ≥ 0.995. The average irradiance over the receiving aperture is given by

$$E(\theta )=E(0)g_{\text{avg}}(\theta ),$$ (D.5)

where *E*(0) is the irradiance over an area of 1 cm^2^,

$$\theta =\tan^{-1}(R/D)$$ (D.6)

is the angle from the center of the lamp to the edge of the receiving aperture, and

$$g_{\text{avg}}(\theta )=1-\frac{\theta }{2{}^{\circ}}\left[1-g_{\text{avg}}(2{}^{\circ})\right].$$ (D.7)

The relative standard uncertainty in irradiance *u*_g_(E)/E due to the goniometric distribution of irradiance is given by

$$\frac{u_{g}(E)}{E}=\frac{E(\theta )-E(0)}{E(0)}=\frac{-\theta }{2{}^{\circ}}\left[1-g_{\text{avg}}(2{}^{\circ})\right].$$ (D.8)

The spectral irradiance of the lamp depends upon the current passing through it. The current is controlled by measuring the voltage across a known resistance and adjusting the output of a power supply to obtain the desired current within a given resolution. From measurements on FEL-type lamps, the relative standard uncertainty in irradiance *u*_i_(*E*)*/E* due to an uncertainty in current *u*(*I*) is given by

$$\frac{u_{i}(E)}{E}=\left(\frac{654.6\;\text{nm}}{\lambda }\right)(0.0006/\text{mA})\;(u(I)),$$ (D.9)

where *λ* is the wavelength. The standard uncertainty of the current arises from both random and systematic effects with assumed Gaussian probability distributions. The uncertainties arising from random effects are the resolution to which the current is set and the uncertainty of the multimeter used to measure the voltage across the resistor. The uncertainties arising from systematic effects are both associated with the resistor: its resistance and temperature coefficient of resistance.

The optic axis for the lamp—receiving aperture combination is centered on the receiving aperture and parallel to its normal. An alignment jig with glass plates parallel to the outside surfaces of the bipost base and marked for the center is used to determine the position of the lamp. The jig is inserted into the lamp mount and the mount is adjusted so that the jig is perpendicular to and centered on the optic axis; the distance from the receiving aperture to the front of the closest plate of the jig is 50.0 cm. The uncertainties in the alignment all arise from systematic effects with assumed rectangular probability distributions. Uncertainties from aligning the jig perpendicular to and centered on the optic axis are equivalent to rotations of the lamp. The irradiance from an ideal lamp would be constant upon these rotations; however, the goniometric distribution of irradiance of actual lamps results in uncertainties. The maximum difference between the goniometric distribution of irradiance at 1° in pitch or yaw to the irradiance at 0° is denoted by *g*_max_, and typically *g*_max_ ≤ 0.01.

When the tripod is used to mount the lamp, the optic axis is defined by the retroreflection of a laser beam from the center of the receiving aperture. An uncertainty in the retroreflection results in an uncertainty in the direction of the normal of the receiving aperture. However, the relative standard uncertainty in irradiance from this effect is essentially zero. The lamp jig is aligned perpendicular to the optic axis by retroreflecting the laser beam. If the uncertainty in the transverse displacement of the retroreflected beam is *u*(*l*_j_), and the distance from laser to the lamp jig is *L*_j_, then the uncertainty in the angle of rotation of the lamp jig *u*(*θ*) is

$$u(\theta )=\tan^{-1}(u(l_{j})/L_{j})$$ (D.10)

When the field calibration unit is used to mount the lamp, the optic axis is defined by the alignment rod. It is assumed that the normal of the receiving aperture is parallel to the long axis of the rod, and *u*(*θ*) is the uncertainty in aligning the face of the lamp jig parallel to the top of the rod. The relative standard uncertainty in irradiance *u*_p_(*E*)/*E* due to an uncertainty in aligning the lamp perpendicular to the optic axis is given by

$$\frac{u_{p}(E)}{E}=\frac{1}{\sqrt{3}}g_{\max}\frac{u(\theta )}{1{}^{\circ}}.$$ (D.11)

The centers of both the lamp and the receiving aperture can be offset from the optic axis. When using the tripod, these are offsets from the laser beam, while when using the field calibration unit these are offsets from the long axis of the alignment rod. If the uncertainty in centering the receiving aperture and the lamp jig on the optic axis are *u*(*l*_a_) and *u*(*l*_j_), respectively, along each axis transverse to the optic axis, then the combined uncertainty *u*(*l*) is

$$u(1)=\sqrt{2}\sqrt{u{\left(l_{a}\right)}^{2}+u{\left(l_{j}\right)}^{2}}$$ (D.12)

and the uncertainty in the angle of rotation of the lamp *u*(*θ*) is

$$u(\theta )=\tan^{-1}(u(l)/D),$$ (D.13)

where *D* is the distance between the receiving aperture and the lamp. The relative standard uncertainty in irradiance *u*_c_(*E*)/*E* due to an uncertainty in centering the receiving aperture and lamp on the optic axis is given by

$$\frac{u_{c}(E)}{E}=\frac{1}{\sqrt{3}}g_{\max}\frac{u(\theta )}{1{}^{\circ}}.$$ (D.14)

The distance between the receiving aperture and the lamp jig is set at 50.0 cm with an uncertainty *u*(*D*). When using the tripod, the distance is set with a stick marked for the correct distance, while with the field calibration unit the top of the alignment rod is the correct distance from the diffuser and the mount is adjusted so that the lamp jig rests on top of the rod. Since the irradiance is proportional to the inverse square of the distance, the relative standard uncertainty in irradiance *u*_d_(*E*)/*E* due to an uncertainty in the distance between receiving aperture and lamp is given by

$$\frac{u_{d}(E)}{E}=\frac{2}{\sqrt{3}}\frac{u(D)}{50\;\text{cm}}.$$ (D.15)

An uncertainty in the wavelength setting of the instrument, arising from a systematic effect with an assumed Gaussian probability distribution, translates into an uncertainty in the measured irradiance. Using the chain rule, the relative standard uncertainty in irradiance *u*_w_(*E*)/*E* due to an uncertainty in wavelength *u*(*λ*) is given by

$$\frac{u_{w}(E)}{E}=\frac{dE}{d\lambda }\frac{u(\lambda )}{E},$$ (D.16)

where d*E*/d*λ* is the derivative of the lamp irradiance with respect to wavelength. The uncertainty in wavelength is a linear fit of the differences between the measured centroids of lines from emission lamps and the actual centroids.

Finally, the uncertainties in the signals arise from random effects and are evaluated using statistical methods.

### D.2. Specific

The sizes of the diffusers are parameters for the uncertainties arising from both the distribution of flux over the diffuser and the goniometric distribution of flux from the lamp. The radii of the diffusers of the instruments are given in Table D.1. For the goniometric distribution, *g*_avg_ = 0.995 was assumed for both lamps, and the distance *D* = 50 cm.

For the lamp current uncertainties arising from random effects, the current was set with a resolution of 0.08 mA, and the relative standard uncertainty of the HP 3457A voltmeter on the 3 V range was 0.0017 % + 6 counts. At a voltage of 0.8 V, the relative standard uncertainty is 2 × 10^−5^, which at 8.0 A corresponds to a standard uncertainty of 0.16 mA. Therefore, the quadrature sum of the standard uncertainty in current arising from random effects is 0.18 mA. For the systematic effects, the relative standard uncertainty in the calibration of the resistor was 16.7 × 10^−6^, which at 8.0 A corresponds to a standard uncertainty of 0.13 mA. The temperature coefficient of resistance of the resistor was 10^−5^/°C. The variation in temperature of the resistor indoors was ± l °C, while the variation outdoors was ± 6 °C, which at 8.0 A corresponds to a standard uncertainty of 0.08 mA indoors and 0.48 mA outdoors. Therefore, the quadrature sum of the standard uncertainty in current arising from systematic effects is 0.15 mA indoors and 0.50 mA outdoors.

For the lamp goniometric distribution, *g*_max_ = 0.01 was assumed for both lamps. When the tripod was used to mount the lamp, the distance from the lamp to the alignment laser was 40 cm. The standard uncertainty in the retroreflection of the laser beam from the lamp jig was 0.2 cm, the standard uncertainty in centering the beam on the diffuser was 0.1 cm for the AEFS and EPA instruments and 0.05 cm for the others, and the standard uncertainty in centering the beam on the lamp jig was 0.05 cm. The standard uncertainty in the distance between the diffuser and the lamp jig was 0.12 cm for the AES and EPA instruments and 0.1 cm for the others. When the field calibration unit was used to mount the lamp, the standard uncertainty in aligning the lamp jig perpendicular to the optic axis was 0.5°, the standard uncertainty in centering the lamp jig was 0.1 cm, and the standard uncertainty in the distance between the lamp jig and the diffuser was 0.1 cm.

The standard uncertainty in wavelength was determined by fitting the centroid differences shown in Fig. 5.4 with straight lines. The wavelength standard uncertainty for the SERC instrument was derived from a determination of this uncertainty for unit UE. The differences at wavelengths shorter than 330 nm determined indoors for the USDA instrument were not used in the fit. The slopes and intercepts of these fits are given in Table D.2.

**Table D.1:** Diffuser radii

Instrument	Radius (cm)
AES	1.60
EPA	1.60
NSF	1.05
SERC	0.95
USDA	1.27

**Table D.2:** Wavelength standard uncertainties

Instrument	Wavelength standard uncertainty	
	Slope (nm/nm)	Intercept (nm)
AES	+ 4.398 3 10^−3^	− 1.357
EPA	− 1.168 3 10^−4^	+ 0.029
NSF	− 5.456 3 10^−4^	+ 0.281
SERC	0	+ 0.050
USDA	− 7.210 3 10^−4^	+ 0.242
